# OFS: on fused SAMI filtered handcraft and DSRSENet features for enhanced skin malignancy detection

**DOI:** 10.3389/frai.2026.1786571

**Published:** 2026-04-29

**Authors:** Shaik Reshma, S. R. Reeja

**Affiliations:** School of Computer Science and Engineering, VIT-AP University, Amaravathi, India

**Keywords:** dual stream residual squeeze excite network, handcraft features, non-handcraft features, optimized feature selection, patient Fitzpatrick skin type, self-attention mutual information

## Abstract

**Introduction:**

Malignant occurrences are increasing in frequency, and skin cancer is emerging as a significant public health concern. Conventional approaches, encompassing comprehensive procedures such as consulting expert opinions, tend to decelerate the therapeutic process.

**Methods:**

A method for classifying skin cancer that incorporates both manually extracted and automatically derived features. This study introduces (i) an innovative method for extracting the feature known as Border Irregularity, which significantly enhances detection capabilities. (ii) This investigation demonstrates that Fitzpatrick skin type significantly influences the detection of cancer. (iii) The Dual Stream Residual Squeeze Excite Network (DSRSENet), a novel deep learning architecture, is employed to extract non-handcrafted features. (iv) Handcrafted features are filtered prior to concatenation with non-handcrafted features through Self Attention-Mutual Information (SAMI) method, which operate based on weighted ranking. (v) A unique optimal feature selection (OFS) method is employed to enhance detection accuracy on concatenated features.

**Results:**

This empirical study is conducted on two publicly available datasets, PAD_UFES_20 and HAM10000, to ensure generalization and robustness. Only handcraft features exhibit detection accuracy of 67.39% and 84.27%, improved to 73.80% and 89.42% with the application of SAMI on both datasets, respectively. After concatenation with the non-handcraft features, OFS further improved the detection accuracy to 93.26% and 95.66% for both datasets respectively, by tuning the hyperparameters precisely.

**Discussion:**

The results demonstrate that Fitzpatrick Skin Type and the proposed Border Irregularity extraction method have a significant impact on skin malignancy detection. Furthermore, the proposed DSRSENet model effectively captures non-handcrafted features, while the SAMI-based filtering and OFS method contribute to improved feature selection. The integration of handcrafted and non-handcrafted features results in superior performance compared to existing methods.

## 1 Introduction

Skin is the protective layer of the body that covers all organs, protecting them from the outside environment. Skin is made up of tissues. Under normal conditions, these tissues behave normally, but in situations like cancer, they will start growing abnormally. Skin cancer originates in the outer layer of the skin and can penetrate deeper layers; in a few situations, it affects the nearby organs, leading to more serious health issues. Like other cancers, skin cancer also has categories. Among the several types of skin cancers, the most dangerous is Malignant melanoma. It is very important to treat this type of malignancy before it spreads. To identify the malignancy of a skin lesion, doctors use the ABCDE (Jütte et al., 2024) method, which is time-consuming. The last term, E, means evolution, which occurs over time. This observation period of evolution causes late detection. Arnold et al. (2022) reported that 325,000 cutaneous melanoma cases were detected and 57,000 life losses were attributed to melanoma in 2020. If this rate remains stable, the melanoma cases will rise to 510,000 and deaths to 96,000 by 2040. This huge loss can be prevented with early detection, which improves the survival rate.

The implementation of an artificial intelligence model enables the replacement of laborious and time-consuming activities. Image processing techniques enable the extraction of handcrafted characteristics, yielding interpretable decision support, including form asymmetry (A), border irregularity (B), color (C), texture (T), and diameter (D). The hormone known as melanin significantly influences the incidence of skin cancer. Excessive melanin synthesis in the body confers resistance against skin cancer. Melanin is the principal component responsible for skin coloring. Individuals with darker skin have a higher relative concentration of melanin than those with lighter skin.

Research shows that people with dark skin have an inbuilt sun protection factor (SPF) of 13.4, whereas people with light skin have an SPF of 3.3 (Gupta et al., 2016). Consequently, it has been shown that persons with darker skin possess a heightened tolerance to UV radiation in comparison to those with lighter skin types, a critical aspect in the etiology of many skin illnesses and cancers (Laikova et al., 2019). According to the study by Marks and Sober (Lim, 2018), the yearly incidence rate of non-melanoma in persons with dark skin is substantially lower, at 1 in 100,000, whereas in individuals with lighter skin, the rate is larger, at 800 in 100,000. An examination of a survey conducted in New Mexico from 1977 to 1978 found a considerable differential in the incidence of basal cell carcinoma, with a ratio of 64.3:495 for those with dark and light skin, respectively. Skin cancer (Narayanan et al., 2010) is the most common type of skin cancer seen in people with light skin, whereas it is less common in people with darker complexions (Agbai et al., 2014). This melanin synthesis directly affects the person's skin type.

**Figure 1:**
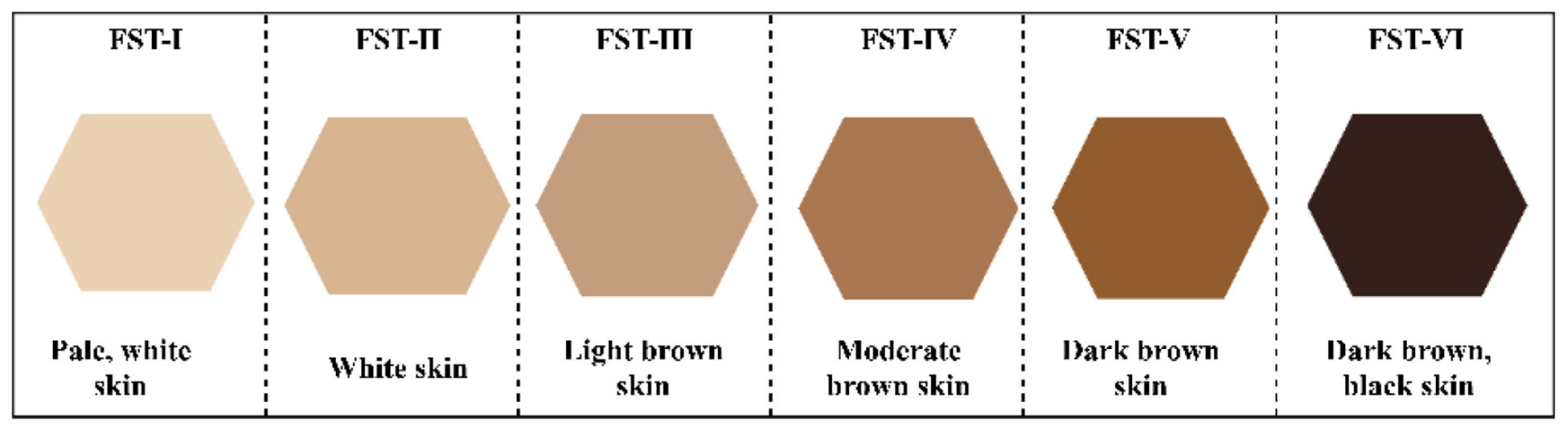
Fitzpatrick skin type pallet (Yu et al., 2024).

The Fitzpatrick skin type (F) was invented in 1975 as a way to measure skin types. This procedure is used to check how dark someone's skin is and how they react to sunlight (Jothishankar and Stein, 2019). It is also used to divide people's skin types into six different groups. Figure 1 shows the six Fitzpatrick skin types and their colors. According to the findings of reference (Sen et al., 2022), persons with lighter skin types, particularly those categorized as F-I, have a greater prevalence of malignant melanoma compared to those with darker skin types, such as F-III and F-IV. The study demonstrated a substantial linear correlation between skin cancer risk and FST, shown by odds ratios of 5.35, 4.58, 2.59, and 1 for F-I, II, III, and IV, respectively (Gupta and Sharma, 2019).

This study integrates the medically proven ABCD components, using Fitzpatrick skin type as an extra custom feature. The neural network generates non-handcrafted characteristics. After the improvement of handmade traits and the addition of non-handcrafted traits. The final set of inputs for the multi-layer perceptron classifier. The hyperparameters are adjusted to improve the accuracy of detecting cancer.

This study can assist dermatologists in identifying skin malignancies. Furthermore, the proposed approach addresses the limitation of requiring dermoscopic devices, as skin lesion images captured using a mobile camera can also be used as input to the model, encouraging teledermatology. The experiments conducted on the PAD-UFES-20 dataset provide evidence that the model can effectively operate on non-dermoscopic images, thereby reducing dependency on specialized imaging equipment.

Major contributions:

- An algorithm has been developed to extract the Border Irregularity (B) component of the ABCD rule, which is a crucial feature for malignancy detection.
- This study exhibits the theoretical and practical significance of the overlooked feature, Fitzpatrick skin type (F), and how it will improve the detection by integrating with other ABCD features.
- A unique weighted-ranking feature selection (SAMI) approach is used to filter handcrafted features that remove noise.
- Non-handcrafted features are derived using the strong and innovative Dual-Stream Residual Squeeze-Excite neural network (DSRSENet), enhancing the entire feature set for classification.
- A unique optimized feature selection (OFS) method is employed to choose features from concatenated handcrafted and non-handcrafted features.
- A Multi-Layer Perceptron (MLP) is employed for the binary categorization of skin lesions into benign and malignant categories based on optimized selected features, yielding good classification accuracy.

## 2 Literature survey

Numerous publications discuss deep learning models for image comparison and the availability of methods for compressing high-quality images. Such compression effectively lowers the computational complexity of classification models (Abdulredah et al., 2024b). Almaraz-Damian et al. (2020) proposed a binary classification method to classify melanoma and nevus types. The proposed CAD system includes preprocessing, feature extraction, and fusion at the last classification pipeline. The ROI for extracting handcrafted features (HF) is obtained through the segmentation process. The prominent HF shape, color, and texture are extracted, and deep learning features are extracted using CNN. The author uses the Mutual Information (MI) measurement to find the relevant features for fusion to increase the detection accuracy. Finally, Linear Regression, SVM, and Relevant Vector Machines (RVM) are used for classification purposes. Among all, RVM gave the best classification performance metrics. The authors used the ISIC2018 dataset for experimentation and ended with 92.4% classification accuracy.

Senan and Jadhav (2021) proposed a system based on the ABCD rule of skin cancer detection. All ABCD features are extracted from the skin lesion images using digital image processing after preprocessing the lesion images. To accurately extract the ABCD features, the images are first passed through a Gaussian filter to remove noise, and morphological operations are applied to enhance the image quality. All the ABCD features are highly dependent upon the contour and ROI. A thresholding technique is used to detect malignancy in the skin lesion. The total Dermoscopic Score (TDS) is used to calculate the total score of malignancy using the ABCD features and their weightage. The images with TDS values greater than 5.64 are considered malignant.

Sharafudeen and Vinod Chandra (2023) proposed a fusion of therapeutic approaches to identify skin lesions. A multi-input, single-output ANN model is used for classification purposes. This model receives handcrafted features, deep learning features, and patient metadata as an input to the multi-model input. This input is processed using various versions of EfficientNet to extract the DL features, while color variegation and GLCM are extracted using handcrafted technique features. After extracting all the features, normalization is applied to prepare them for the ANN classifier. The main techniques used are Majority voting and weighted majority voting by estimating the model weights using a grid search model.

Kumar et al. (2024) proposed that the extraction of handcrafted features from various domains will be provided to a multithreaded CNN for classification. The datasets HAM10000 and Dermnet were used for assessment, yielding accuracies of 89.71% and 88.57%, respectively. Michela Effendie and colleagues (Effendie and Aguiar-Pulido, 2024) proposed a fusion approach to integrate dataset modalities using Vision Transformers, with the extracted features being classified through XGBoost. Experimentation conducted on the PAD_UFES_20 dataset yielded an accuracy of 86%, a precision of 85%, a recall of 86%, and an F1-score of 85%.

De Lima Mota et al. (2025) introduced two deep learning models, namely ResNet and ViT. The experimentation carried out on PAD_UFES_20 achieved a malignancy detection accuracy of 80.63% and an F1 score of 79.89%. Mendes et al. (Mendes and Krohling, 2022) presented a combinatorial feature set that integrates features derived from CNN, manually constructed components, and patient clinical data. Handcrafted characteristics include ABCD and texture. ResNet50 was utilized to extract the CNN features. The experimentation conducted on the PAD_UFES_20 dataset yielded an accuracy of 86.1% in cancer detection.

Bansal et al. (2022) presented a comprehensive feature set that combines handcrafted elements with deep learning models. Shape, color, and texture are extracted using handcrafted methods, while EfficientNet and ResNet are used for deep learning feature extraction. All the features have now been integrated and passed to the ANN classifier. The experimentation was conducted using two datasets: HAM10000 and PH2. High accuracies of 94.9% for HAM10000 and 98% for PH2 have been recorded for the integration of the feature set. For classifying skin lesions, Abdulredah et al. (2024a) suggested combining a customized CNN AD_Net model with a pretrained VGG16. The input images were compressed using the Huffman method before being sent to the models. The ISIC2019 image dataset was used to test this model, and the accuracy was 99.18%. To detect skin cancer, Dhibar (2024) developed a deep autoencoder model using ResNet101. Machine learning and deep learning models were combined to create the deep autoencoder. The ISIC2019 dataset is used for experimentation, yielding an accuracy of 96.03%. Abdulredah et al. (2025) suggested using a SkinWiseNet to identify skin cancer. The model uses multiple routes to extract features from the input photos. Four datasets are used to test the model. Of the four datasets, the Melanoma Skin Cancer dataset had the highest accuracy (99.86%), while the HAM10000 dataset had the lowest accuracy (81.93%). Table 1 summarizes the existing works.

**Table 1:** Summary of existing works.

References	Handcrafted features (HF)	Deep learning model, methodology	Classifier and type of classification	Dataset	Result
(Almaraz-Damian et al. 2020)	Shape, color, texture	MobilenetV2, Fusion of features using MI metric	Relevant vector machine, Binary classification	ISIC2018	Accuracy = 92.4%
(Senan and Jadhav 2021)	Asymmetry, border, color, diameter	NA, TDS value	TDS score, binary classification	PH2	Accuracy = 84%
(Sharafudeen and Vinod Chandra 2023)	Color, texture	EfficientNetB4 to B7, multi-input and single-output model	ANN, multiclass classification	ISIC2018, ISIC2019	Balanced accuracy = 91.93%, 94.13%
(Kumar et al. 2024)	Image, Spectrogram, Cepstrum domain	NA	Multithreaded CNN, multiclass classification	HAM10000	Accuracy = 89.71%
(Effendie and Aguiar-Pulido 2024)	NA	ViT	XGBoost, multiclass classification	PAD_UFES_20	Accuracy = 86%
(De Lima Mota et al. 2025)	NA	ResNet and ViT	ViT, multiclass classification	PAD_UFES_20	Accuracy = 80.63%
(Mendes and Krohling 2022)	ABCD + texture	Resnet50	CNN, binary classification	PAD_UFES_20	Accuracy = 86.1%
(Bansal et al. 2022)	Shape, color, and texture	EfficientNet, ResNet	CNN, binary classification	HAM10000	Accuracy = 94.9%
(Dhibar 2024)	NA	Autoencoder+ResNet101	Multiclass classification	ISIC2019	Accuracy = 96.03%
(Abdulredah et al. 2025)	NA	SkinWiseNet	Binary classification	Melanoma skin cancer, HAM10000	Accuracy = 99.86%, 81.93%

### 2.1 Border irregularity

Malignant skin lesions often exhibit irregular boundaries. To identify irregularities in the borders of skin lesions, Ali et al. (2020b) employed a thresholding strategy that combined a Convolutional Neural Network (CNN) and the Gaussian naïve Bayes algorithm. The CNN uses image smoothing, canny edge detection, convexity, Zernike moments, and fractal dimension to determine the likelihood of border irregularity. The Gaussian naïve Bayes algorithm is applied to convexity, Zernike moments, and fractal dimension. Classification is performed using a threshold value after combining the probabilities from the Convolutional Neural Network (CNN) and Gaussian Naïve Bayes models.

Abeysinghe and Sotheeswaran (2020) introduced two techniques, namely the distance difference method and the gradient approach, for detecting border irregularity. These methods include traversing along the continuous boundary of the lesion. Threshold values are used to categorize skin lesions in both scenarios. Ali et al. (2020a) employed the concept of convexity from segmented masks and fractal dimension from fuzzy edge images to compute the prediction probability. Finally, they applied a threshold to categorize the border irregularity. Kurachi et al. (2015) used curvature to calculate the border irregularity of skin lesions. This curvature and shape asymmetry is then utilized to classify the irregularity of the lesion's border.

## 3 Methodology

**Figure 2:**
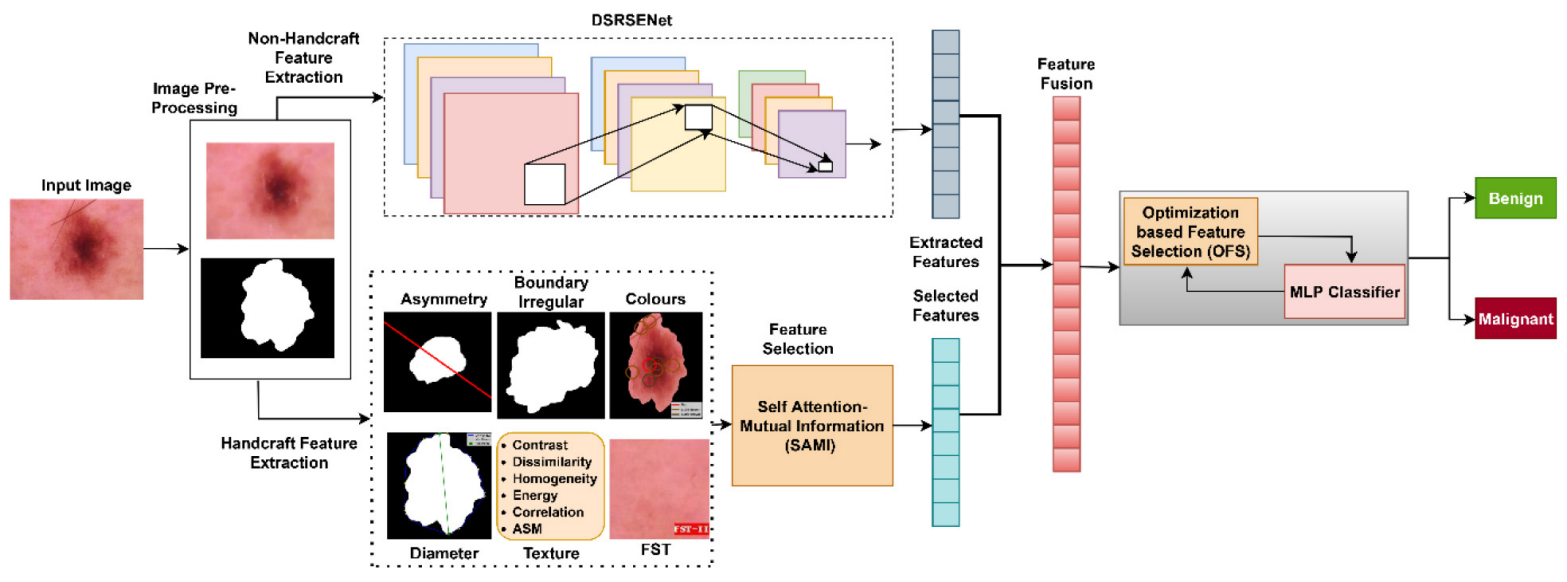
Complete architecture of the system.

The proposed approach for classifying skin lesion images as benign or malignant is depicted in Figure 2. The approach employs optimized feature selection for several characteristics collected from skin lesion images. Two kinds of feature extraction are employed. Handcrafted characteristics, including ABCD and F. All handcrafted features are evaluated and filtered utilizing the feature selection model SAMI. Non-handcrafted characteristics are retrieved via a combination of shallow and deep network paths, using a DSRSENet. Both handcrafted and non-handcrafted characteristics are meticulously developed and picked using an optimized feature selection approach to enhance detection accuracy.

### 3.1 Preprocessing

Image preprocessing is implemented as the initial phase, during which three primary operations are conducted on the images. The initial action is resizing; all images are adjusted to a uniform dimension of 256 x 256. The subsequent application of a dull razor technique is utilized on the photos to eliminate hair-like artifacts. By following a sequence of image processing steps, converting to a grayscale image, applying blackhat and thresholding, and finally applying the inpaint image processing technique, an artifact-removed image is produced. The binary mask of the skin lesion is retrieved using the widely utilized medical image segmentation model UNET. The model is trained on HAM10000 images and used to predict the binary mask for the PAD_UFES_20 dataset, as there is no ground truth binary mask.

### 3.2 Handcrafted features extraction

#### 3.2.1 Border irregularity (B): novel method

A unique method is proposed to ascertain border irregularity by computing the area discrepancy. This procedure is a contrastive evaluation method. An approximately defined uneven border for a skin lesion is referred to as a serrated contour. The actual boundary of the lesion has been assessed in relation to the serrated contour by calculating the area difference between the two.

As an initial stage, the binary mask contour (*C*) of the segmented lesion is identified as represented in Equation 1.


$$\begin{array}{l} C=\left\{\left(x_{1},y_{1}\right),\left(x_{2},y_{2}\right),\ldots \ldots \left(x_{n},y_{n}\right)\right\} \end{array}$$ (1)

Using an ellipse fit technique, the contour is analyzed to identify the points where two shapes overlap due to border irregularities and collect the intersecting points (*I*) are computed using Equations 2 and 3.


$$\begin{array}{l} I_{i}=\;\left\{\left(x_{i},y_{i}\right)\;|\;C\left(x_{i},y_{i}\right)=\;\varepsilon \left(x_{i},y_{i}\right)\right\} \end{array}$$ (2)

$$\begin{array}{l} I=\;\left\{I_{1,}\;I_{2},\;\ldots \ldots \ldots \ldots I_{n}\right\} \end{array}$$ (3)

Where *C*(*x*_*i*_, *y*_*i*_) is a point on the curve and ε(*x*_*i*_, *y*_*i*_) is a point on the ellipse drawn. *I*_*i*_(*x*_*i*_, *y*_*i*_) is the point of intersection of the curve and the ellipse.

The angles (θ) formed by the crossing points is measured at the central point of the contour using Equation 4, and a mean angle (μΘ) is computed using these measurements using the Equation 5.


$$\begin{array}{l} \Theta_{i}=\tan^{-1}\left(\frac{y_{c}-y_{i}}{x_{c}-x_{i}}\right) \end{array}$$ (4)

Where (*x*_*c*_, *y*_*c*_) = *c* (centroid)


$$\begin{array}{l} \mu \Theta =\;\overset{n}{\underset{i=1}{\sum }}\Theta_{i} \end{array}$$ (5)

To mark the places on the binary mask contour that have a difference in mean angle, straight lines (*y*−*y*_*c*_) are drawn from the centroid with the mean angle difference, as defined in Equation 6.


$$\begin{array}{l} y-y_{c}=tan\left(\mu \Theta \right)\left(x-x_{c}\right) \end{array}$$ (6)

The intersection points (S) between binary mask contour and radial lines drawn from the centroid are identified, as defined in Equations 7 and 8.


$$\begin{array}{l} S_{i}=\;\left(x_{i},y_{i}\right) \end{array}$$ (7)

$$\begin{array}{l} S=\;\left\{S_{1,}S_{2},\ldots \ldots \ldots \ldots S_{n}\right\} \end{array}$$ (8)

Join all the points to form a serrated contour (S), as defined in Equation 9.


$$\begin{array}{l} S_{c}=\left\{\left(x_{1},y_{1}\right),\left(x_{2},y_{2}\right),\ldots \ldots \left(x_{n},y_{n}\right)\right\} \end{array}$$ (9)

The freshly produced serrated contour indicates irregularity in the border. Using the serrated contour as a reference, the quality of the binary mask contour is computed. This feature is quantified by comparing the area of both contour sections as defined in Equation 10.


$$\begin{array}{l} \Delta A=\;A_{c}-\;A_{s} \end{array}$$ (10)

Where *A*_*c*_—Area of contour, *A*_*s*_—Area of serrated contour.

**Figure 3:**
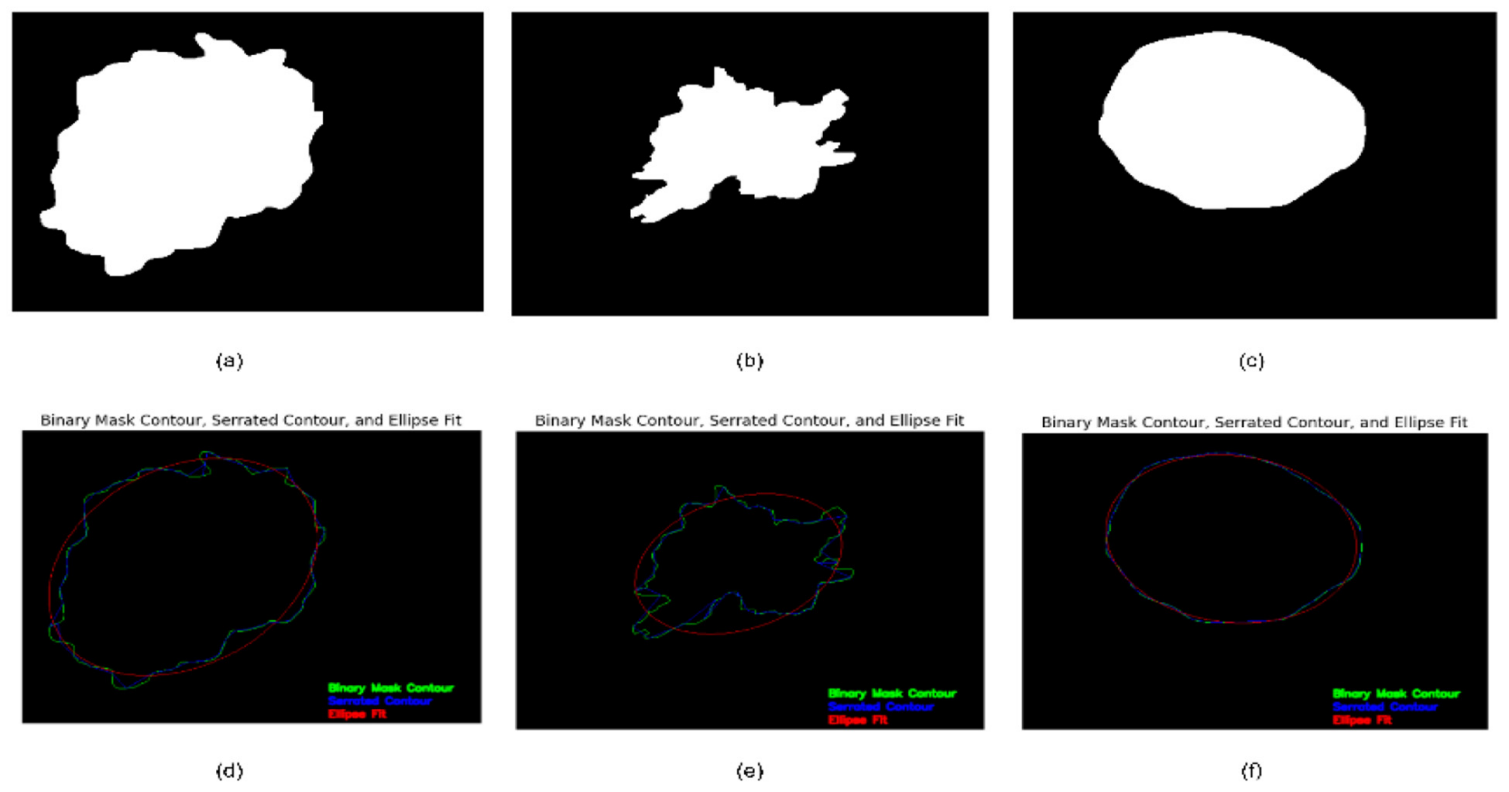
Novel border irregularity check method **(a–c)** binary mask of lesions, **(d–f)** binary mask contour, serrated contour, and ellipse fit.

A k-means clustering approach is employed to categorize lesions into regular and irregular borders. The unsupervised clustering techniques utilize the area difference computed by the suggested approach, in conjunction with other essential properties such as shape descriptors, fractal dimension, and Zernike moments. Algorithm 1 represents the steps followed for the construction of the serrated contour, followed by calculating the area difference. Figure 3 illustrates the novel method to draw a serrated contour.

Algorithm 1 **Algorithm for area difference between binary mask and serrated contour.**

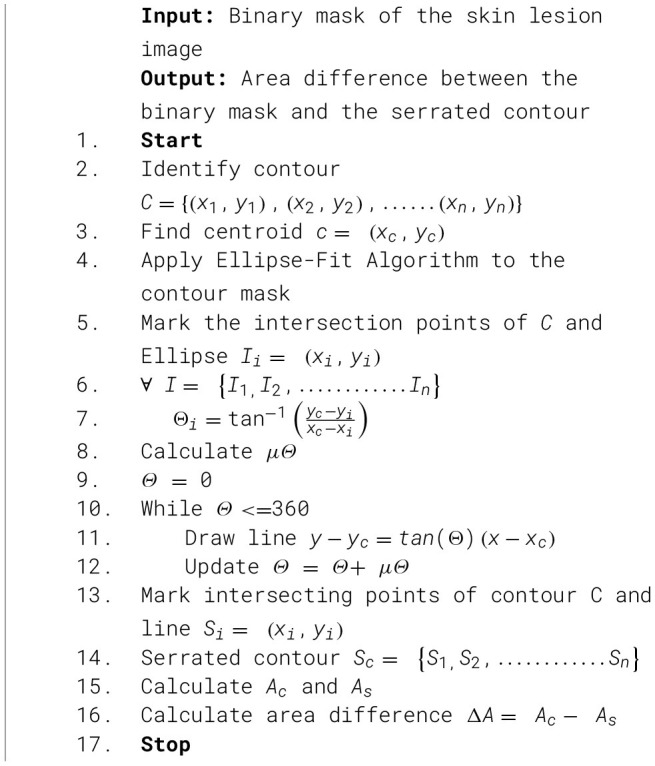

#### 3.2.2 Other handcrafted features

Additional handcrafted characteristics encompass asymmetry, color variations, texture, diameter, and Fitzpatrick skin type classification.

##### 3.2.2.1 Asymmetry

Asymmetry (A) of lesion shape significantly influences the detection. Malignant lesions typically exhibit asymmetry, whereas benign lesions tend to be symmetrical. The asymmetry of the lesion shape has been verified using the binary mask of the lesion. Initially, the centroid of the lesion shape is identified using an ellipse-fitting technique. Subsequently, a straight line is drawn through the identified centroid. Additional parallel lines have been drawn alongside the straight line to encompass the entire lesion area. There is a midpoint for each line, and these midpoints are connected to make a symmetric axis. These midpoints do not form a straight line *y* = *Cx*+*D* altogether, as the lesion shapes are irregular, a deviation of a few midpoints is observable. Mean deviation is calculated from the mean distances of deviated midpoints from the straight symmetric axes, as defined in Equation 11. For lesion shape rotations of 180 degrees, deviation errors are calculated, and the mean deviation error (μdE) is found. A threshold of 5 is used based on the mean deviation error value. Mean deviations over 5 are considered asymmetric, and those below 5 are considered symmetric.


$$\begin{array}{l} \mu_{dE}=\;\sqrt{\frac{\overset{n}{\underset{i=1}{\sum }}{\left(y_{i}-D-Cx_{i}\right)}^{2}}{n}} \end{array}$$ (11)

##### 3.2.2.2 Color

Color (C) plays a significant role as per the ABCD rule. The more colors appear on the lesion, the more concerning it is. The ABCD rule defines specifically six colors of lesions. The threshold values for each color are defined in Equations 12 to 17, as follows:


$$\begin{array}{l} Black=\;\sum \left(R<0.2\;\&G<0.2\;\&B<0.2\right) \end{array}$$ (12)

$$\begin{array}{l} Red=\;\sum \left(R<0.8\;\&G<0.2\;\&B<0.2\right) \end{array}$$ (13)

$$\begin{array}{l} White=\;\sum \left(R>0.8\;\&G>0.8\;\&B>0.8\right) \end{array}$$ (14)

$$\begin{array}{l} Bluegray=\;\sum R<0.2\;\left(0.32<G<0.72\right)\\ \;\&\left(0.34<B<0.74\right) \end{array}$$ (15)

$$\begin{array}{l} Lightbrown=\;\sum \left(0.6<R<1\right)\;\left(0.32<G<0.72\right)\;\\ \;\&\left(0.05<B<0.45\right) \end{array}$$ (16)

$$\begin{array}{l} Darkbrown=\;\sum \left(0.2<R<0.6\right)\;\&\left(0.06<G<0.46\right)\;\\ \;\&\left(0<B<0.33\right) \end{array}$$ (17)

Where R, G, and B represent the colors Red, Green, and Blue.

##### 3.2.2.3 Diameter

Diameter (D) evaluation is significant because it directly correlates with the severity of the lesion. The diameter is determined using the segmentation mask of the lesion. The Euclidean distance between the points on the contour that are 180 degrees apart is considered the diameter of the lesion. Equation 18 represents the formula for calculating Euclidean distance.


$$\begin{array}{l} Diameter=\;\sqrt[{2}]{{\left(i-k\right)}^{2}+{\left(j-l\right)}^{2}} \end{array}$$ (18)

Where (*i, j*) and (*k, l*) are the two points on boundary that differ by a 180-degree angle.

##### 3.2.2.4 Texture

Texture (T) of the lesion presents a notable feature that may help distinguish malignant lesions. The lesion's texture is obtained using the GLCM method. Equations 19 to 23 defines the extraction of GLCM features.


$$\begin{array}{l} Contrast=\;\overset{n-1}{\underset{x=0}{\sum }}\overset{n-1}{\underset{y=0}{\sum }}{\left(x-y\right)}^{2}P_{x,y} \end{array}$$ (19)

$$\begin{array}{l} Dissimilarity=\;\overset{n-1}{\underset{x=0}{\sum }}\overset{n-1}{\underset{y=0}{\sum }}|x-y|P_{x,y} \end{array}$$ (20)

$$\begin{array}{l} Homogeneity=\;\overset{n-1}{\underset{x=0}{\sum }}\overset{n-1}{\underset{y=0}{\sum }}\frac{P_{x,y}}{1+|x-y|} \end{array}$$ (21)

$$\begin{array}{l} Energy=\;\overset{n-1}{\underset{x=0}{\sum }}\overset{n-1}{\underset{x=0}{\sum }}{\left(P_{x,y}\right)}^{2} \end{array}$$ (22)

$$\begin{array}{l} Correlation=\;\overset{n-1}{\underset{x=0}{\sum }}\overset{n-1}{\underset{y=0}{\sum }}\frac{\left(1-u_{x}\right)\left(1-u_{y}\right)P_{x,y}}{\sigma_{x}\sigma_{y}} \end{array}$$ (23)

Where (*x, y*) are coordinates, *P*_*x, y*_ is the normalized gray level co-occurrence probability between *x*, *y* gray levels. *u*_*x*_ and σ_*x*_ are the mean and standard deviation values of *P*_*x*_, and *u*_*y*_ and σ_*y*_ are the mean and standard deviation values of *P*_*y*_. Equations 24 to 27 defines the calculation of the mean and variance.


$$\begin{array}{l} \mu_{x}=\;\overset{n-1}{\underset{x,y=0}{\sum }}x\;P_{x,y} \end{array}$$ (24)

$$\begin{array}{l} \mu_{y}=\;\overset{n-1}{\underset{x,y=0}{\sum }}y\;P_{x,y} \end{array}$$ (25)

$$\begin{array}{l} {\sigma_{x}}^{2}=\;\overset{n-1}{\underset{x,y=0}{\sum }}P_{x,y}{\left(x-\mu_{x}\right)}^{2} \end{array}$$ (26)

$$\begin{array}{l} {\sigma_{y}}^{2}=\;\overset{n-1}{\underset{x,y=0}{\sum }}P_{x,y}{\left(y-\mu_{y}\right)}^{2} \end{array}$$ (27)

##### 3.2.2.5 Fitzpatrick skin type

Fitzpatrick skin type (F) is an additional characteristic of the patient that has a high impact on the development of skin cancer. The non-lesion area of the skin lesion image is identified to extract the skin type. The process will begin by dividing the image into four equal sections, then subdividing one of those sections into an additional four parts. The image of the unaffected skin of the patient is extracted in this manner. The RGB image has now been converted into the CIELab color space, and its luminance (L), along with the chromatic components (a, b) has been extracted. The Individual Typological Angle (ITA) is calculated using the formula provided in Equation 28. Finally, based on the threshold values in Table 2 (Krishnapriya et al., 2021), the Fitzpatrick skin type is extracted for the patient.


$$\begin{array}{l} ITA=arcTan\left(L-\frac{50}{b}\right)*\left(\frac{180}{\pi }\right) \end{array}$$ (28)

**Table 2:** Threshold values for fitzpatrick skin type.

ITA(i)	Fitzpatrick skin type
*i*°> = 50	F-I
25° < = *i* < 50	F-II
0° < = *i*° < 25°	F-III
−25° < = *i*° < 0°	F-IV
−50° < = *i*° < −25°	F-V
*i*° < −50°	F-VI

### 3.3 Non-handcraft features extraction

**Figure 4:**
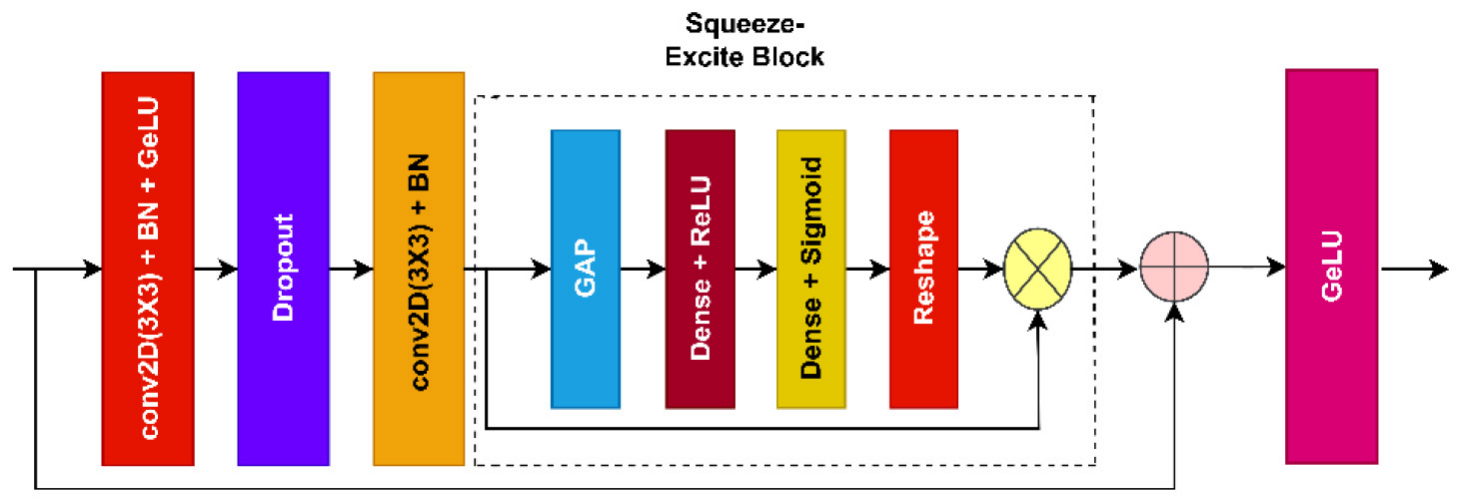
RSE block.

**Figure 5:**
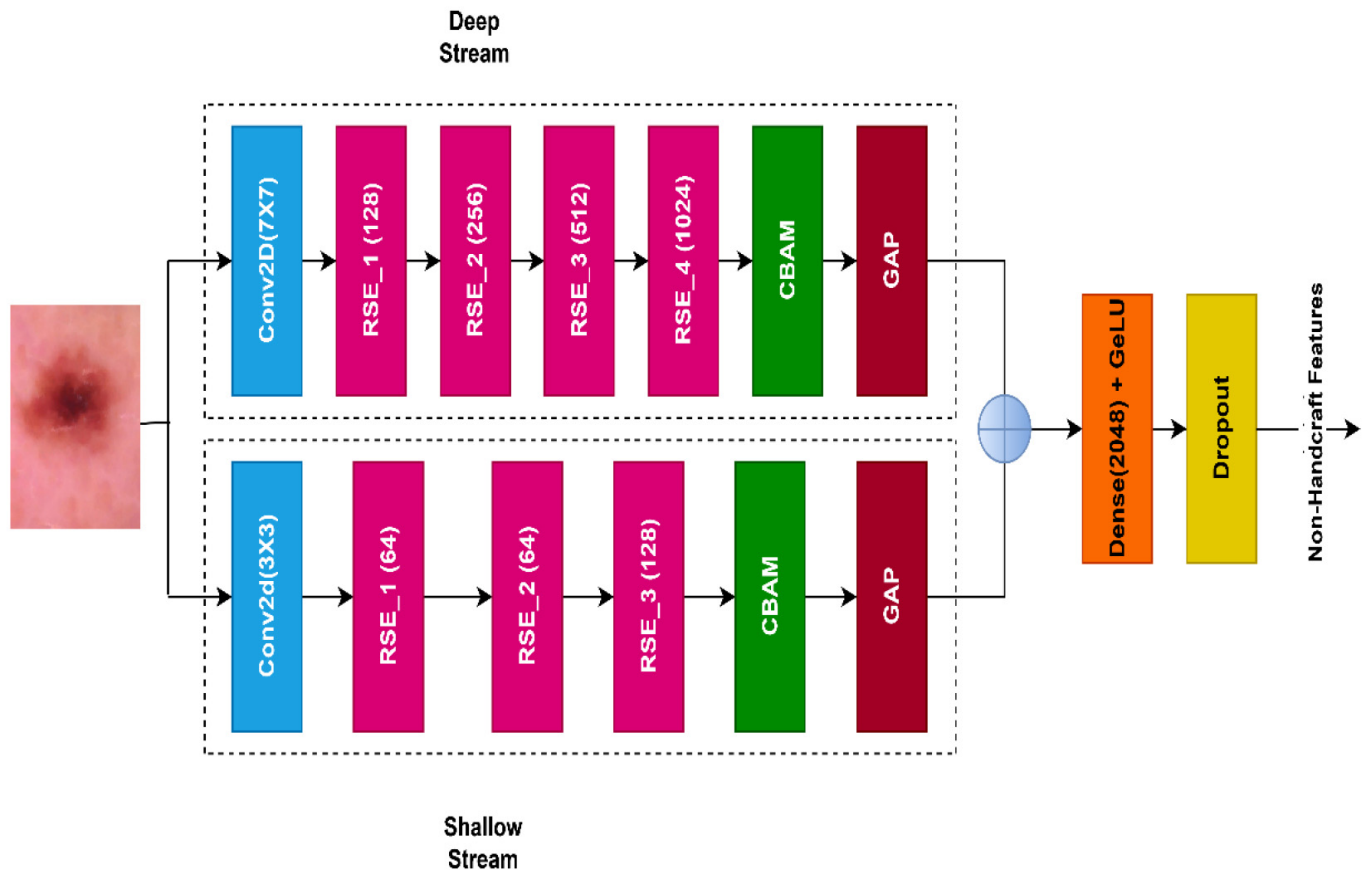
DSRSENet Architecture.

A customized CNN model, known as the dual stream residual squeeze excite network (DSRSENet), integrates a dual-input stream residual network with a squeeze-excite block to extract non-handcrafted features. The DSRSENet accepts the input of 299 299 3, height, width, and channels, respectively, are determined by the model complexity and original input image resolution. The net is built on the backbone of integration of the residual block and squeeze-excite block (RSE). Figure 4 represents the RSE block layer architecture. The net processes input from two different streams: one acts as a shallow stream, and the other acts as a deep stream to extract the features. Figure 5 represents the DSRSENet architecture layers. Finally, both the streams are concatenated to get more refined features.

The shallow stream is followed by a convolutional layer with 3 3 kernel of 64 filters. The output of the first conv layer is forwarded to the first RSE block. The RSE block starts with a convolution layer with 3 3 kernel followed by batch normalization and the GeLU activation function. A dropout is applied on this layer, and the forwarded output is sent to the second convolutional layer with 3 3 kernel, followed by batch normalization. The output of the second conv layer is given to the Squeeze-Excite block (SE). A residual skip connection is implemented here by adding the output of the SE layer and the input of the RSE block, and GeLU activation is applied on the final addition. Equation 29 represents the RSE layer.


$$\begin{array}{c} RSE\left(x_{i+1}\right)=f\left(x\right)*\emptyset \left(f\left(x\right)\right) \end{array}$$ (29)

Where $\emptyset \left(x\right)=\;\frac{1}{2}\;\left[1+err\left(\frac{x}{\sqrt{2}}\right)\right]$, *err*(*x*) is the error function.


$$\begin{array}{c} f\left(x\right)=concatenation\left(x,g\left(x\right)\right) \end{array}$$ (30)

g(x)=SE(C_BN(Droupout(GeLU(C_BN(x)))) (31)

Equation 30 represents the concatenation process and Equation 31 represents the sequence of layers in the RSE block. Where *C*_*BN*(*x*) = *BN*(*Conv*(*x*)), *BN*(*x*) represents the batch normalization, *Conv*(*x*) represents the convolution operation. *Droupout*(*x*) is the dropout layer,*GLU*(*x*) = x*∅(*x*) activation function.


$$\begin{array}{c} SE\left(x\right)=mul\left(x,\frac{1}{1+e^{h\left(x\right)}}\right) \end{array}$$ (32)

$$\begin{array}{c} h\left(x\right)=flatten\left(ReLU\left(flatten\left(GAP\left(x\right)\right)\right)\right) \end{array}$$ (33)

Equation 32 represents the outcome of SE block and Equation 33 represents the sequence of layers in SE block. where *flatten*(*x*) represents the dense layer of 1D representation, *ReLU*(*x*) = *max*(0, *x*) represents the activation function, *GAP*(*x*) represents the Global average pooling operation.

Likewise, the shallow stream contains three RSE blocks in sequence with three different filters, 64, 64, and 128, respectively, and ends with a Convolutional Block Attention Module (CBAM) block. The purpose of adding CBAM is to suppress the irrelevant features by enhancing the important regions of where to focus and what to look for.

The deep stream starts with a convolutional layer with 3 × 3 kernel of 64 filters with a stride of 2. Four RSE blocks with filter sizes of 128, 256, 512, and 1,024, respectively, follow. The deeper layers of the deep stream help extract more fine-grained features compared to the shallow stream. At the end, a CBAM layer is applied to fine-filter the important features among the extracted ones. Before concatenation, both the stream outputs are passed to global average pooling layers individually. After the concatenation, a dense layer with 2,048 filters and a GeLU activation function is applied. The dense layer with an activation function helps in learning non-linear, complex relations among the features.

### 3.4 Feature selection and classification

Handcrafted and non-handcrafted elements have been extracted from identical input photographs, while the extraction methodologies vary. Handcrafted features are based on statistical and feature-related characteristics, and the extraction methods used are insufficient to enhance the obtained values for the final application. There is a considerable probability of noise integration. Conversely, non-handcrafted features obtained from deep convolutional layers improve the extracted features more effectively, thereby reducing the probability of noise inclusion in these features. This justifies executing feature selection on the manually produced features before concatenation. The efficacy of non-handcrafted features may be compromised if handcrafted elements are included without adequate noise removal.

#### 3.4.1 Self-attention mutual information

A weighted ranking feature selection approach, termed SAMI, is employed on handcrafted characteristics to identify the most significant features. SAMI integrates self-attention and mutual information techniques. The retrieved handcrafted characteristics from the skin lesion images may comprise highly correlated, irrelevant, noisy, redundant, and non-contributory data. The SAMI feature selection approach is employed on the handcrafted features to eliminate duplicated, strongly correlated, and non-contributory characteristics. The self-attention mechanism used for the handcrafted features aids in managing the dependencies among those features.

Mutual information mitigates statistical noise by assigning rankings relative to the goal values. This helps identify unnecessary characteristics and redundancy by calculating mutual information among all features. The SAMI feature selection assigns weights by including the mutual information value into the self-attention of the feature before applying the activation function. Equation 34 defines SAMI, Equation 35 defines the handcrafted feature set, Equations 36 to 38 define the self-attention parameters, and Equation 39 defines the mutual information.


$$\begin{array}{c} SAMI=softmax\left(\frac{QK^{T}}{\sqrt{d_{k}}}+\;I\left(x,y\right)\right)*V \end{array}$$ (34)

$$\begin{array}{ccc} f_{h}\; & \in & \;HCF^{n*d} \end{array}$$ (35)

$$\begin{array}{c} Q=\;f_{h}W^{Q} \end{array}$$ (36)

$$\begin{array}{c} K=\;f_{h}W^{K} \end{array}$$ (37)

$$\begin{array}{c} V=\;f_{h}W^{V} \end{array}$$ (38)

$$\begin{array}{c} I\left(x,y\right)=\;\underset{x\in X}{\sum }\underset{y\in Y}{\sum }p\left(x,y\right)\;\log\left(\frac{p\left(x,y\right)}{p\left(x\right)p\left(y\right)}\right) \end{array}$$ (39)

HCF comprises a collection of handcrafted features, with *f*_*h*_ is one of these characteristics. The attention scores, attention weights, and final self-attention of the feature *f*_*h*_ are computed as follows. Where *p*(*x, y*) is joint probabilities of features x, y and *p*(*x*)*p*(*y*) is the marginal probability.

#### 3.4.2 Optimized feature selection

The SAMI manages the intra-type redundancy inside the handcrafted features. Following the amalgamation of handcrafted and non-handcrafted features, additional issues arise, including cross-model correlation and scale imbalance due to the predominance of non-handcrafted feature density. A novel OFS tackles this issue more effectively by utilizing a relevance score to remove redundancy and amplify the impact of important characteristics in classification.

Using mutual information (MI), variance (*v*), and redundancy (Rd), a relevance score (R) is determined. This acts as a fitness function. The MI helps in finding out the relation between the features, and *v* assists in discovering significant features. Rd negatively impacts the model, since an overabundance of duplicate characteristics might confuse it, reducing its ability to identify cancerous skin lesions. The redundant features are identified using the correlation coefficient, and their contribution to classification is minimized. Finally, the top k features are selected based on the R values calculated for each feature.

Accuracy is chosen as the objective function. High classification accuracy is achieved by tuning the parameters α, β, γ, and k. The hyperparameters α, β, and γ are utilized for managing the weights of statistics MI, *v*, and Rd, respectively. The weight values will range from 0 to 1. The range of k values will be established from 2 to the total number of features extracted.

During the tuning process of the α, β, and γ hyperparameters, initial values will be assigned randomly. In subsequent iterations, these values will be adjusted using the specified updating equations, which will account for changes in accuracy. The k value, which represents the top-k features, is updated via random search. The values will be modified according to the objective function.


$$\begin{array}{c} f=Concatenate\left(f_{h},f_{nh}\right) \end{array}$$ (40)

$$\begin{array}{c} R_{i}=sigmoid\left({\alpha *MI}_{i}+\beta *v_{i}-\gamma *Rd_{i}\right) \end{array}$$ (41)

Equation 40 represents the concatenation of both handcrafted and non-handcrafted features and Equation 41 represents the relevance score formula. Where *f*_*h*_ are the handcrafted features, *f*_*nh*_ are non-handcrafted features, and both features are concatenated to form the final feature vector *f*. $MI\left(x,y\right)=\;\underset{x\in X}{\sum }\underset{y\in Y}{\sum }p\left(x,y\right)\;log\left(\frac{p\left(x,y\right)}{p\left(x\right)p\left(y\right)}\right)$, $v\left(x\right)=\frac{1}{n}\;\overset{n}{\underset{i=1}{\sum }}{\left(x_{i}-\;\mu_{x}\right)}^{2}$, μ_*x*_ is the mean and $Rd\left(x,s\right)=\;\underset{y\varepsilon s}{\max}|corr\left(x,y\right)|$, $corr\left(x,y\right)=\;\frac{cov\left(x,y\right)}{\sigma \left(x\right)\sigma \left(y\right)}$. The updating function used is


$$\begin{array}{c} \delta^{t+1\;}=\;\delta^{t}+\Delta \;*\;Acc\;*\;\Delta \delta \end{array}$$ (42)

Equation 42 represents the parameter update function. Where η = 0.01 and Δ*Acc* = *Acc*^*t*^− *Acc*^*t*−1^, Δδ = δ^*t*^− δ^*t*−1^ and δ = {α, β, γ}. *Acc* is the model's accuracy on the test dataset. Algorithm 2 represents the step-wise manner of OFS implementation.

Algorithm 2 **Algorithm for OFS.**

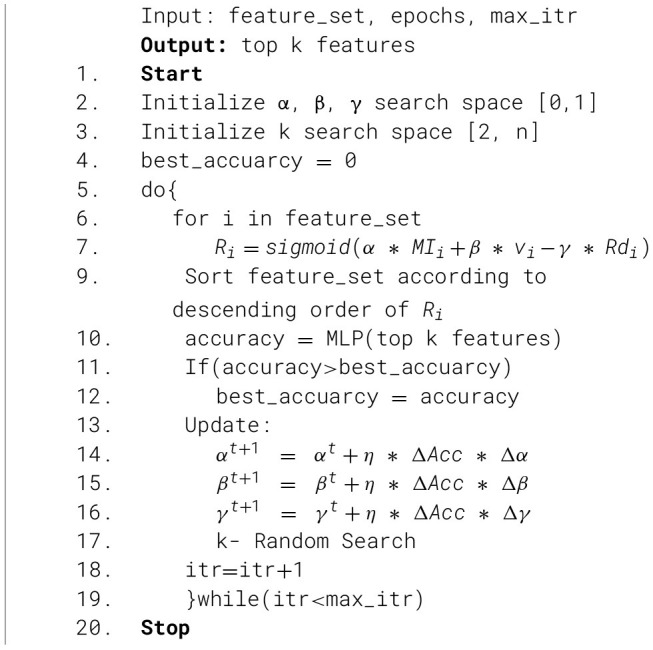

#### 3.4.3 Classification

The training model utilized is a Multi-Layer Perceptron (MLP). The MLP demonstrates efficacy in comprehending non-linear relationships between features to improve classification outcomes. The final feature set is processed through a three-layer deep MLP consisting of 128, 64, and 32 neurons in each respective layer, utilizing the ReLU activation function. Each layer is regularized through the application of batch normalization and dropout techniques to mitigate the risk of model overfitting. The final output layer consists of a dense layer with a sigmoid activation function for the classification of skin lesion images as benign or malignant categories. Algorithm 3 represents the entire process of the proposed malignancy detection.

Algorithm 3 **Algorithm for classification.**

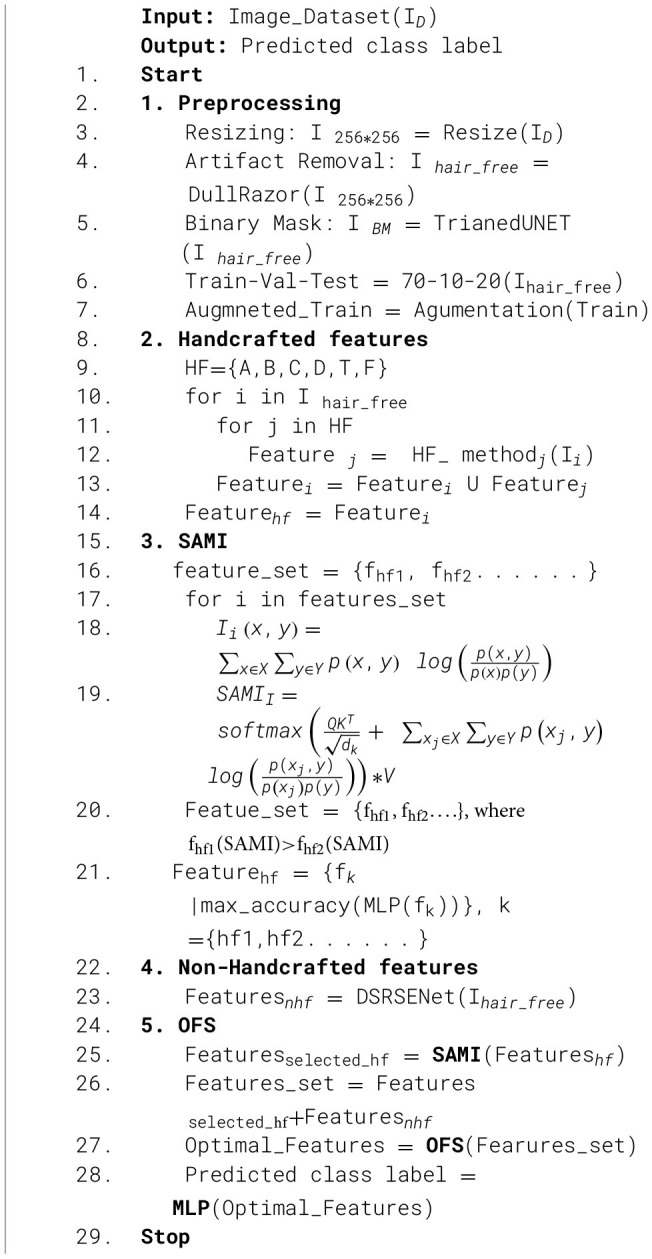

## 4 Model evaluation and experimental results

### 4.1 Dataset and augmentation

**Figure 6:**
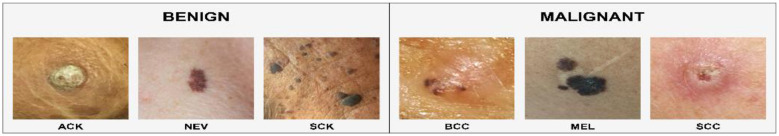
PAD_UFES_20 sample images.

**Figure 7:**
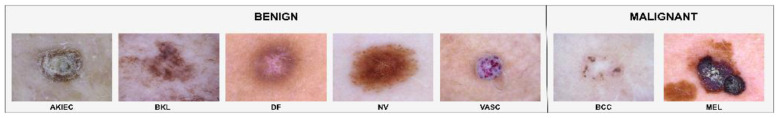
HAM10000 sample images.

**Figure 8:**
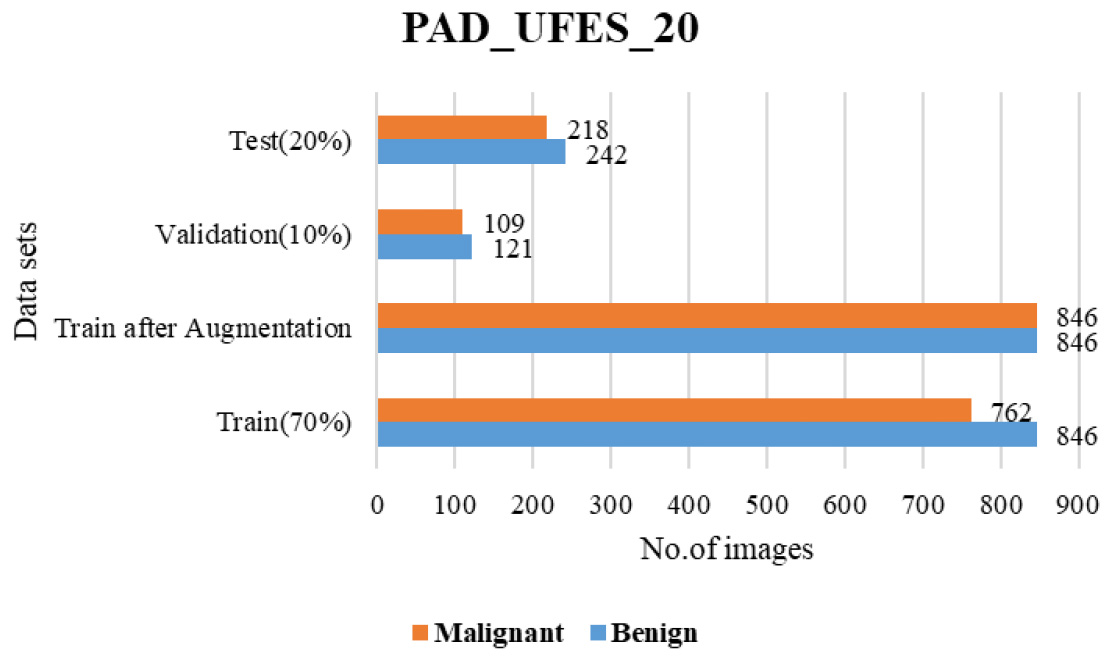
Train-validation-test sets of PAD_UFES_20.

**Figure 9:**
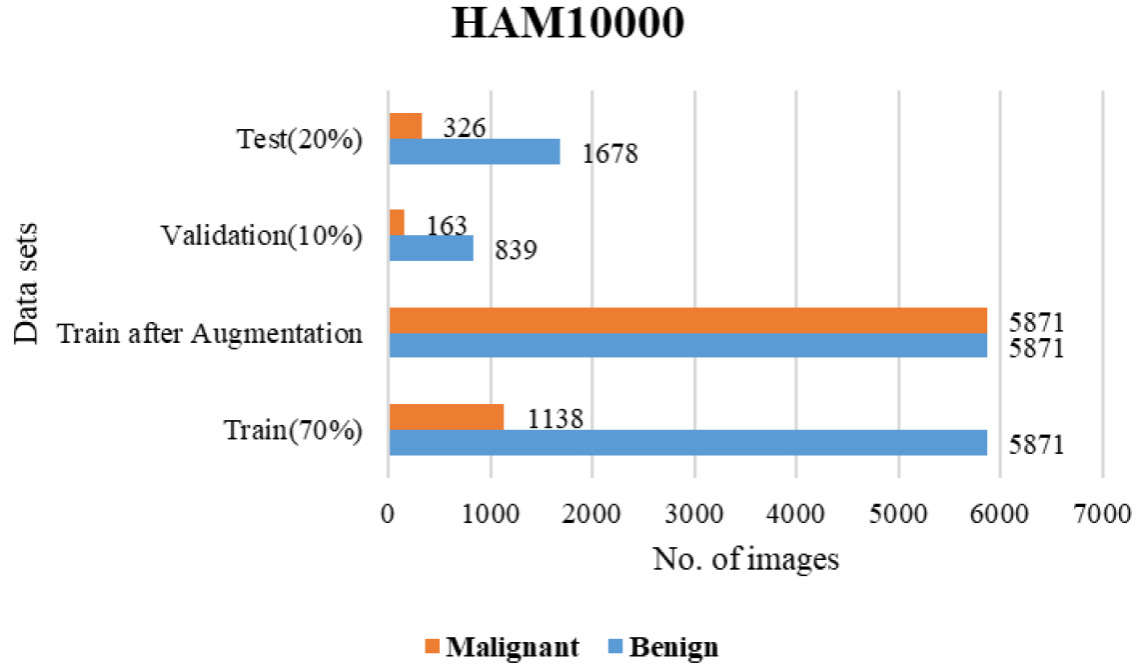
Train-validation-test sets of HAM10000.

The present work leverages two well-acknowledged skin cancer datasets, specifically PAD_UFES_20 (Magalhães et al., 2025) and HAM10000 (Khan et al., 2024). The collection PAD_UFES_20 has 2,298 photos of skin lesions. This collection of photos is categorized into 6 groups, with 3 representing malignant conditions and 3 representing skin problems. The HAM10000 group consists of 10,015 photos distributed across seven categories, with two being malignant and the other four being skin illnesses. The dataset is transformed into a binary class by classifying instances as either malignant or non-cancerous. Both PAD_UFES_20 and HAM10000 are currently classified as either benign or malignant. Figures 6, 7 show the sample skin lesions of both datasets. For training, validation, and testing, these datasets are divided into a 70-10-20 ratio. Every experiment carried out in this work uses the same 70-10-20 split ratio. 20% of the test set is used to test the model's performance, 10% is used for validation and early stopping based on validation accuracy, and 70% of the training set is used for training. Data augmentation technology balances the training datasets before they are entered into the classifier. Rotation, zooming, and flipping with relation to the left and right are used for data augmentation. Zoom and flip are performed with up to 30% probability, and rotation is performed with up to 70% probability. The sample information for the test, validation, and training sets before and after augmentation approaches to address data imbalance is shown in Figures 8, 9.

### 4.2 Experimental setup

The tests are performed in Python 3.10 using the Keras, Scikit-learn, and OpenCV libraries on an Intel Xeon CPU with 64 GB of RAM and an NVIDIA GeForce GTX 1080 Ti GPU card.

### 4.3 Performance metrics

The effectiveness of the proposed DL and optimizer combination model classification is tested using four metrics: accuracy, F1-score, precision, and recall, as defined in Equations 43 to 46.


$$\begin{array}{c} Accuracy=\;\frac{T\text{\_}p+T\text{\_}n}{T\text{\_}p+T\text{\_}n+F\text{\_}p+F\text{\_}n} \end{array}$$ (43)

$$\begin{array}{c} F_{1}-SCORE=\;\frac{2*T\text{\_}p}{2*T\text{\_}p+F\text{\_}p+F\text{\_}n} \end{array}$$ (44)

$$\begin{array}{c} Precision=\;\frac{T\text{\_}p}{T\text{\_}p+F\text{\_}p} \end{array}$$ (45)

$$\begin{array}{c} Recall=\;\frac{T\text{\_}p}{T\text{\_}p+F\text{\_}n} \end{array}$$ (46)

### 4.4 Performance analysis of handcraft features

#### 4.4.1 Impact of novel border irregularity algorithm

**Figure 10:**
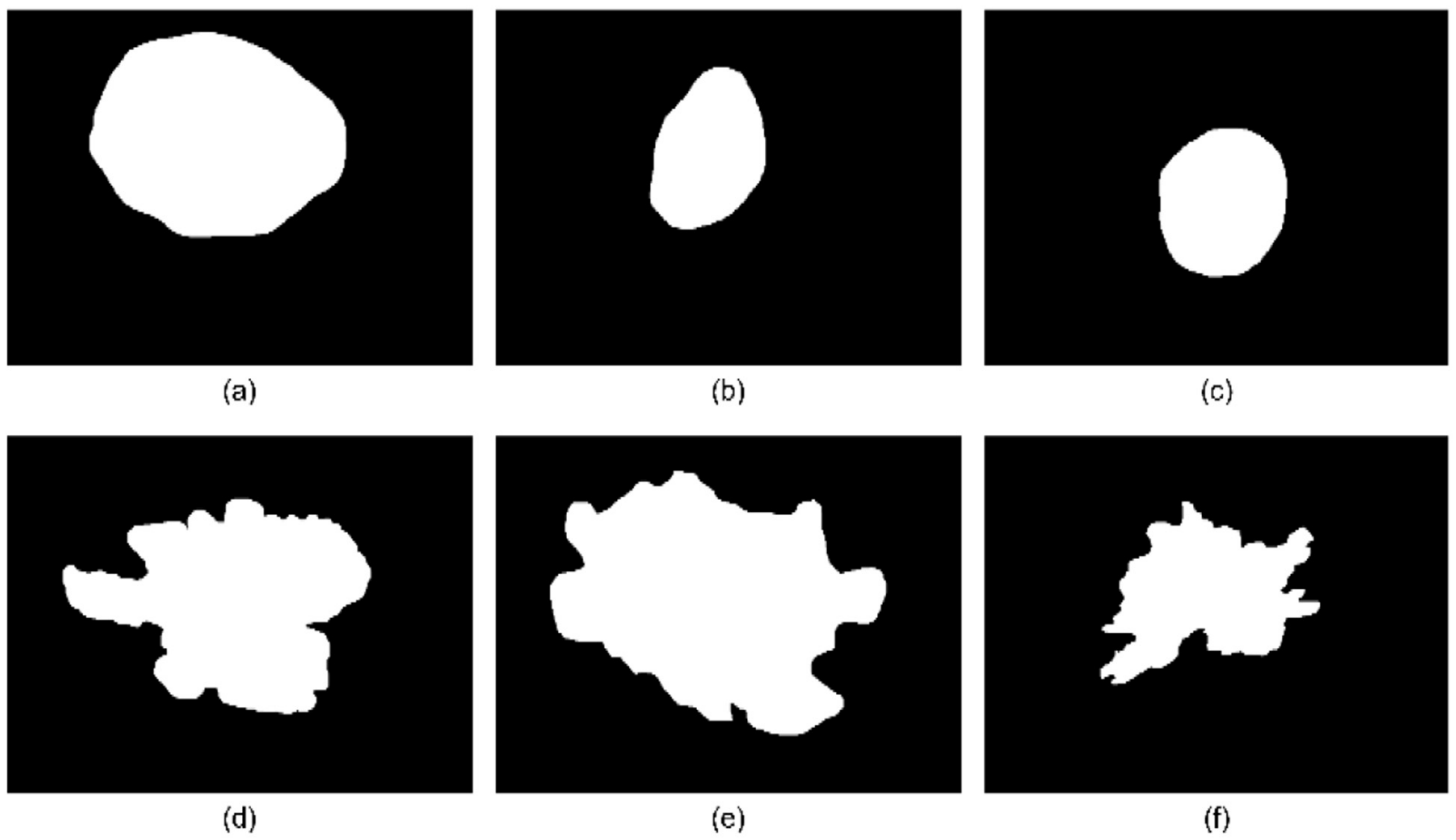
Classification result of border irregularity feature. **(a–c)** are sample images classified as border regular shapes, **(d–f)** are sample images classified as border irregular shapes using the Novel Border Irregular algorithm.

The novel combinatorial method has been developed to leverage the area discrepancy between the actual border and serrated contour, in conjunction with fractal dimensions, Zernike moments, and other shape descriptors, to effectively categorize lesion types into two classifications: regular boundary shapes and irregular boundary shapes. Figure 10 presents a typical outcome of the classification of border irregularity. The evolution of the border irregularity feature is conducted through disease classification, as ground truth data for this feature is unavailable. The identical MLP architecture with a depth of three layers is employed for disease classification, both with and without feature use. The evaluation of the proposed serrated contour area difference is conducted both with and without its application, and compared with the overall disease classification. Table 3 illustrates the assessment of the border irregularity feature alongside the proposed serrated contour area difference border irregularity algorithm, using overall disease classification as a reference.

**Table 3:** Performance efficiency of novel border irregularity on skin cancer classification.

Dataset	Features	Accuracy (%)	Precision (%)	Recall (%)	F1-score (%)	AUC
PAD_UFES_20	HCF(ACDTF) + B	59.57	59.58	59.57	58.79	0.62
	HCF(ACDTF) + novel proposed B algorithm	67.39	67.47	67.39	67.14	0.72
HAM10000	HCF(ACDTF) + B	83.77	70.18	83.77	76.38	0.59
	HCF(ACDTF)+ novel proposed B algorithm	84.27	81.50	84.27	78.45	0.68

Hcf, handcraftED features; a, asymmetry; b, irregular border (without area difference); c, colors; d, diameter; t, texture.

#### 4.4.2 Impact of Fitzpatrick skin type feature

The assessment of the Fitzpatrick skin type feature is conducted by juxtaposing disease classification outcomes with and without the inclusion of the feature. Table 4 provides a comprehensive overview of the performance metrics. It is clearly observed that the involvement of the Fitzpatrick skin type feature increased the detection accuracy of skin cancer.

**Table 4:** Performance efficiency of Fitzpatrick skin type.

Features	Accuracy	
	PAD_UFES_20	HAM10000
HCF(ABCDT)	56.52	83.92
HCF(ABCDFT)	67.39	84.27

Hcf, handcraftED features; a, asymmetry; b, irregular borders (novel proposed); c, colors; d, diameter; t, texture; f, Fitzpatrick skin type.

### 4.5 Performance analysis of non-hand-crafted features

**Figure 11:**
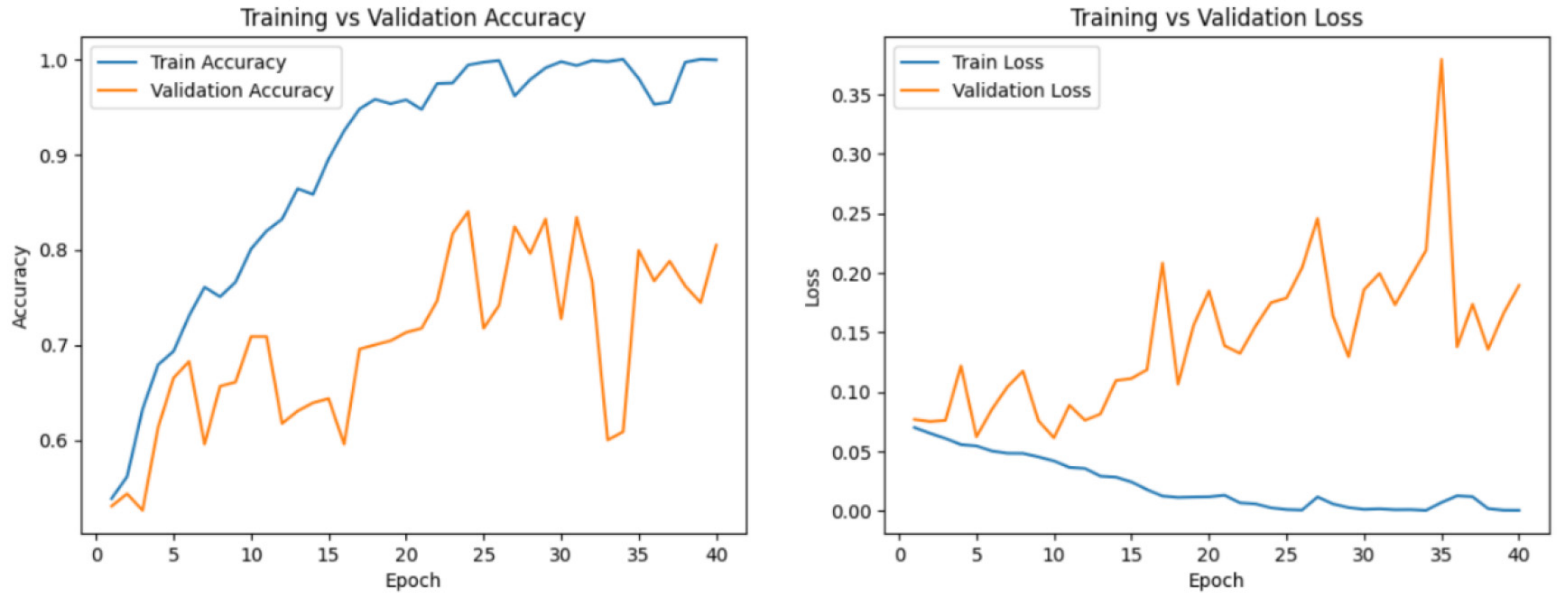
Train-validation accuracy and loss curve of DSRSENet on PAD_UFES_20.

**Figure 12:**
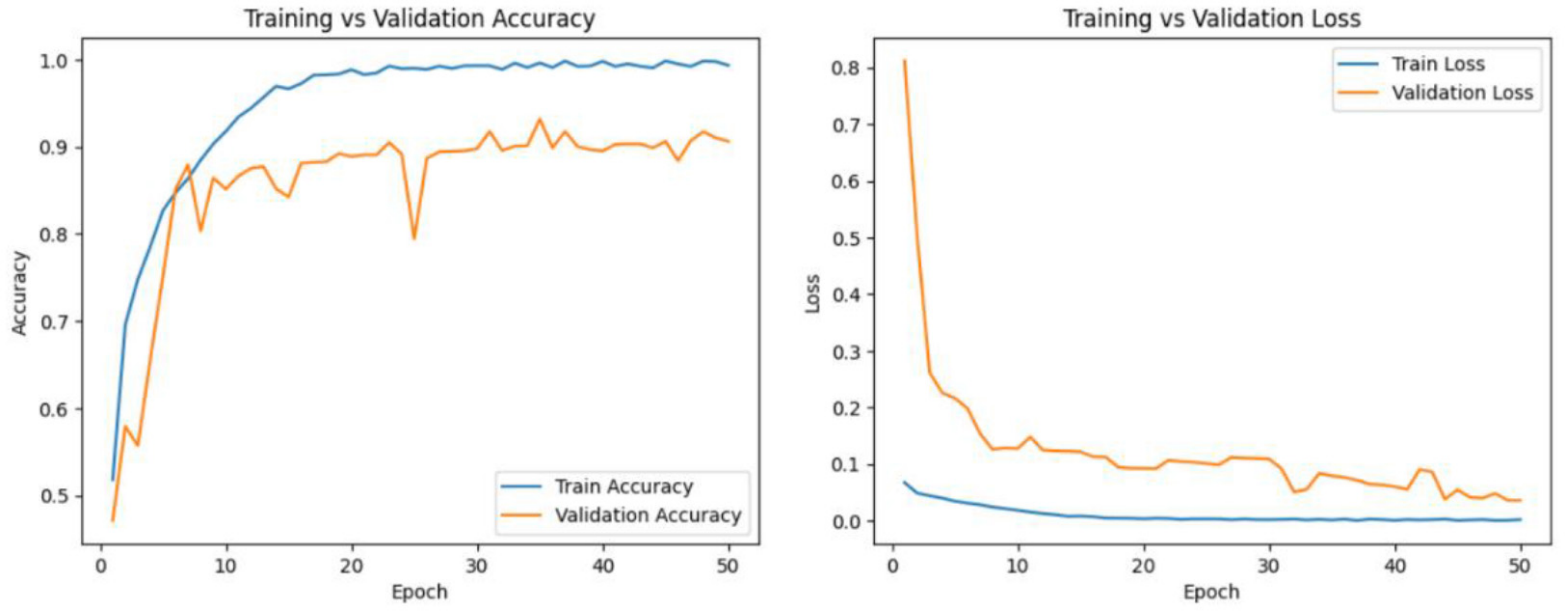
Train-validation accuracy and loss curve of DSRSENet on HAM10000.

**Figure 13:**
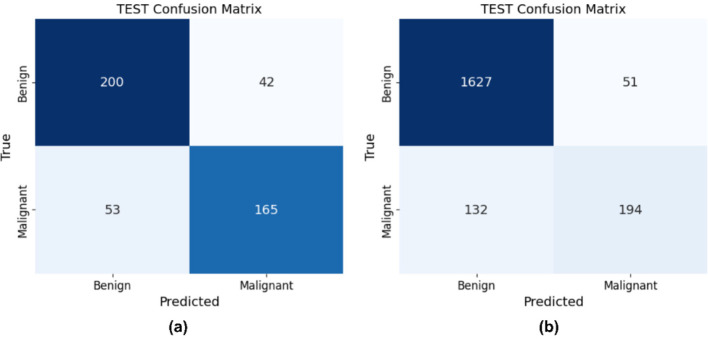
Confusion matrix of DSRSENet on **(a)** PAD_UFES_20 **(b)** HAM10000 test sets.

DSRSENet is implemented on preprocessed datasets and extracts non-handcrafted features from the input images. In the implementation of the RSE block, the default stride is set to 1, and the dropout rate is established at 0.2. For the SE block within the RSE, a default ratio of 16 is maintained to ensure optimal balance. The model is trained for 100 epochs, incorporating early stopping with a patience of 15 based on validation accuracy. The hyperparameters used during implementation include the Adam optimizer with a learning rate of 0.001, a batch size of 32, a dropout rate of 0.3, and a sigmoid activation function. Binary cross-entropy is used as the loss function, with an input size of 299 × 299 × 3 for skin lesion images. Figures 11, 12 represent the train-validation accuracy and loss graphs of the network, and Figure 13 represents the confusion matrix of the model on both datasets. Table 5 represents the DSRSENet results for both datasets.

**Table 5:** Performance metrics of DSRSENet on both datasets.

Dataset	Accuracy	Precision	Recall	F1-score
PAD_UFES_20	79.35	79.36	79.35	79.31
HAM10000	90.87	90.33	90.87	90.33

The DSRSEnet model is a dual-stream architecture comprising 23.7 million trainable parameters. The model achieves an average inference time of 0.1315 sec/image and 7.59 frames per second. The computational complexity of the model is 79.47 GFLOPs per inference, indicating the high computational cost. These heavy computations are managed with the high-performance GPU systems.

### 4.6 Feature concatenation and classification

#### 4.6.1 Result analysis of SAMI

**Figure 14:**
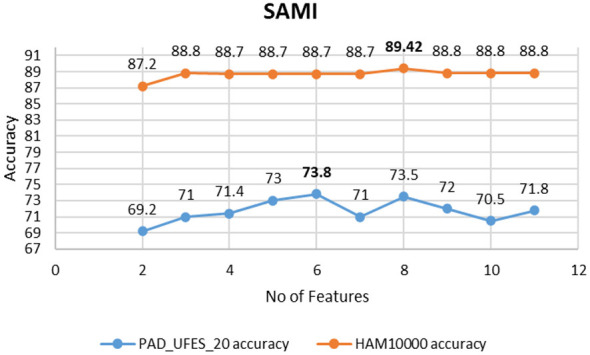
Accuracy vs. features graph for SAMI-weighted ranking feature selection.

The detection of the disease is significantly influenced by the features provided to the model. The most effective features contribute to enhanced detection accuracy. The features chosen by the SAMI undergo evaluation using the MLP model. A 5-fold cross-validation method is used, with the final accuracy determined as the average of the accuracies from all 5 folds. The feature selection algorithm operates based on the fundamental principle of the select-k best algorithm. Accuracy is an objective for selection in the select-k best algorithm. The classification model is supplied with the top k features derived from the SAMI best methodology. Figure 14 illustrates the graph depicting mean accuracy in relation to the number of features selected by the feature selection method. The analysis indicates that selecting the top 6 features yields the highest classification accuracy for PAD_UFES_20, which includes asymmetry, border irregularity, colors, correlation, ASM, and Fitzpatrick skin type. For HAM10000, the top 8 features that achieve the highest accuracy are asymmetry, border irregularity, color, diameter, contrast, homogeneity, energy, and Fitzpatrick skin type. Table 6 illustrates the accuracy of disease detection utilizing only handcrafted features. It highlights the effect of SAMI feature selection by comparing detection performance metrics prior to and following the application of SAMI on the handcrafted features.

**Table 6:** Classification accuracy before feature selection and after feature selection using SAMI.

Features	Accuracy	
	PAD_UFES_20	HAM10000
Before SAMI	68.04	84.87
After SAMI	73.80	89.42

#### 4.6.2 Result analysis of OFS

The optimized feature selection method is employed to enhance hyperparameter tuning for optimal feature extraction utilizing a neural network. The OFS is implemented with 50 iterations and an early stopping criterion, utilizing a patience of 15 based on validation set accuracy. The hyperparameters used during implementation include the Adam optimizer with a learning rate of 0.01, a batch size of 32, and a sigmoid activation function. Binary cross-entropy is used as the loss function.

Among the 11 handcrafted features, the SAMI filtering method selected 6 features for the PAD-UFES-20 dataset and 8 features for the HAM10000 dataset. In parallel, DSRSENet is used to extract 2048 deep features from each dataset. These deep features are concatenated with the selected handcrafted features, resulting in a combined feature vector of 2054 features for PAD-UFES-20 and 2056 features for HAM10000. Finally, OFS-based feature selection is applied to the concatenated feature set to identify the most discriminative features for classification.

**Figure 15:**
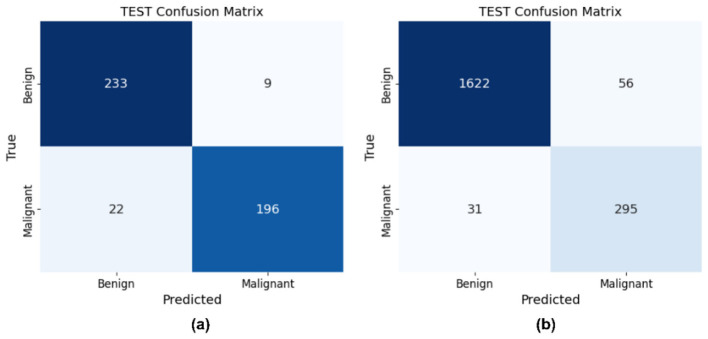
Confusion matrix of OFS on **(a)** PAD_UFES_20 **(b)** HAM10000.

**Figure 16:**
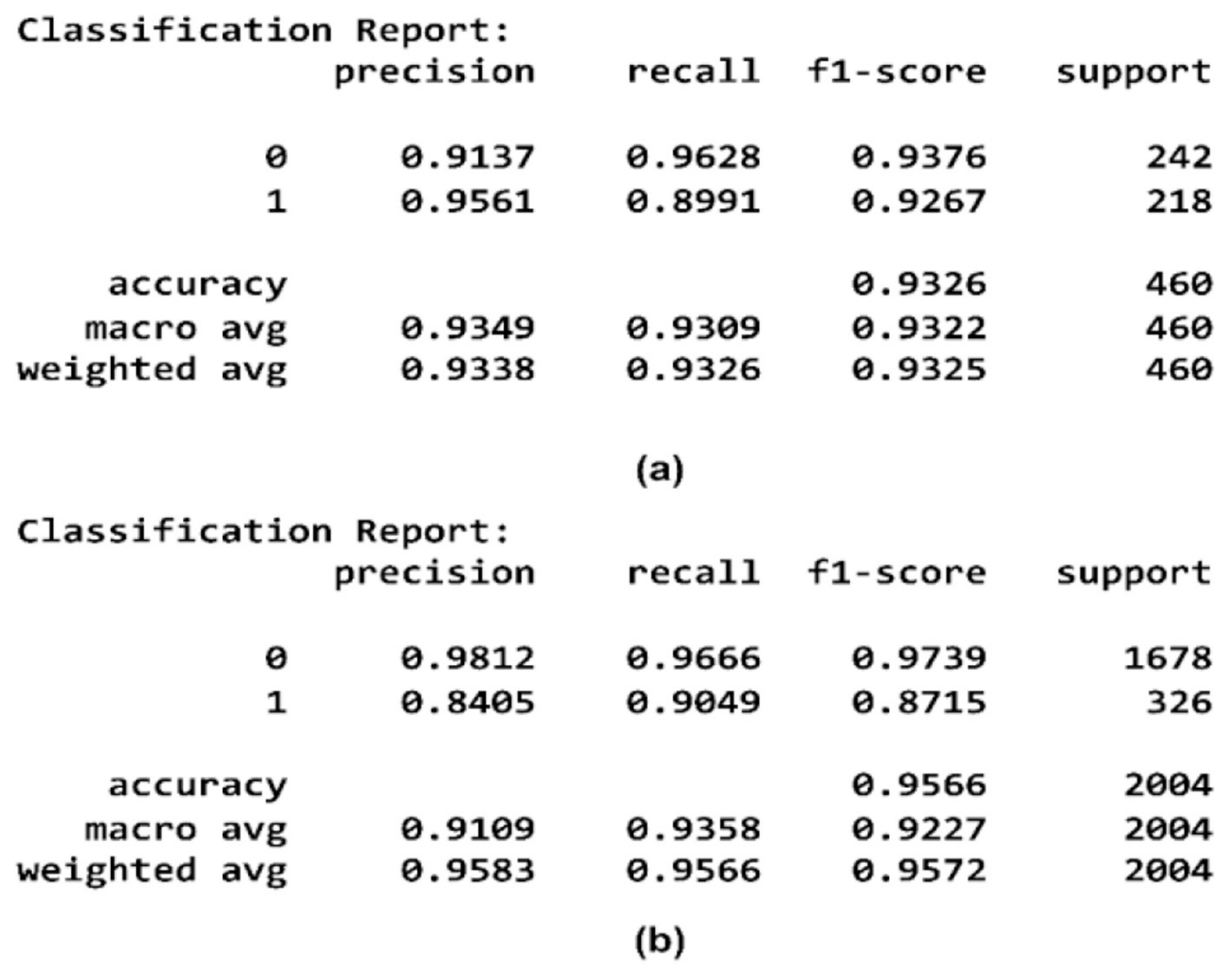
Classification reports of OFS on **(a)** PAD_UFES_20 **(b)** HAM10000.

**Figure 17:**
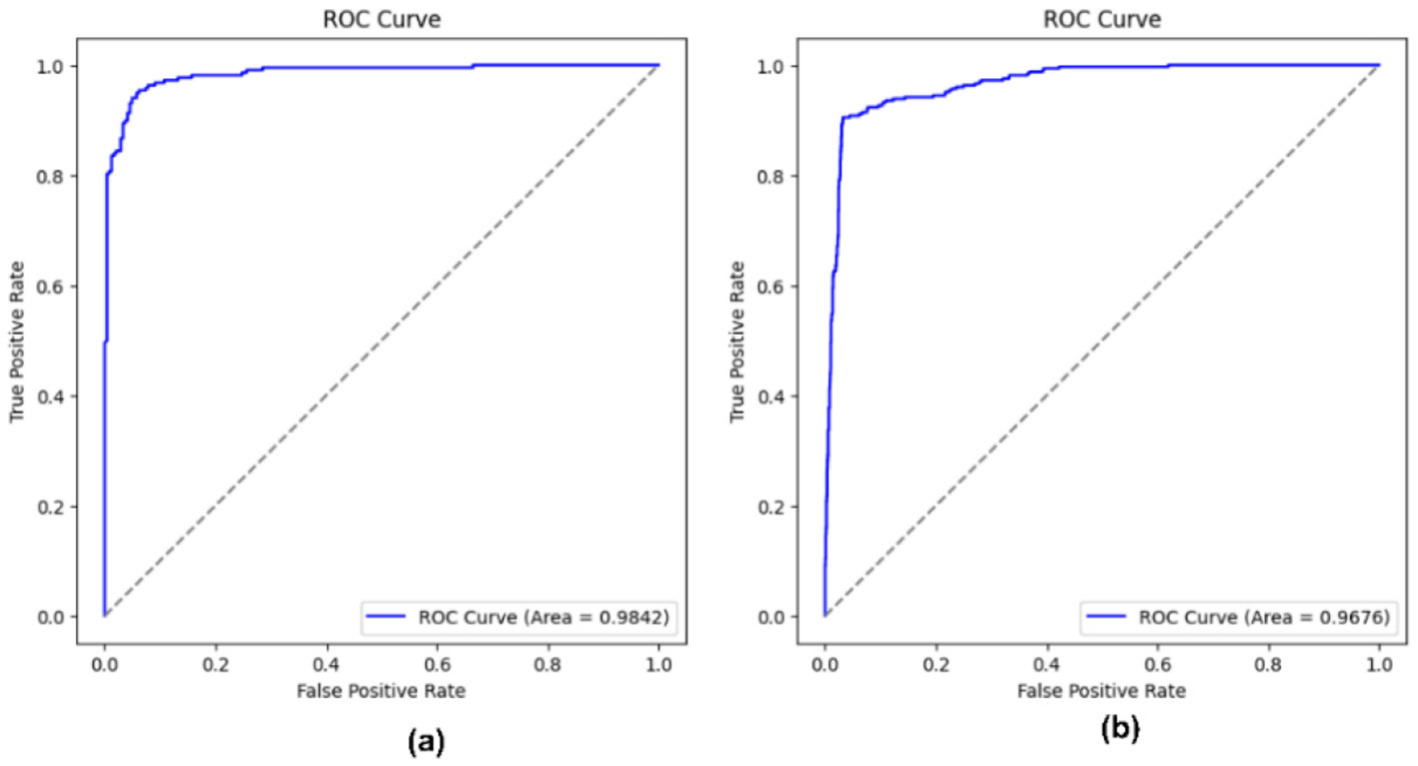
ROC curve of OFS on **(a)** PAD_UFES_20 **(b)** HAM10000.

The implementation on the PAD_UFES_20 dataset yields a high test accuracy of 93.26% with the hyperparameters set to α = 0.6396, β = 0.02503, γ = 0.2753, and utilizing *k* = 1261 features. The HAM10000 dataset, with hyperparameters values α = 0.6398, β = 0.02509, γ = 0.2751, and *k* = 825, achieved a high-test accuracy of 95.66%. The resultant values of hyperparameters α, β, and γ for both datasets are very similar, ensuring that the selected features are informative and non-redundant. This consistency across both datasets validates the effectiveness of the proposed OFS. Figures 15–17 illustrate the performance metrics of the model across both datasets.

### 4.7 t-SNE visualization of OFS

**Figure 18:**
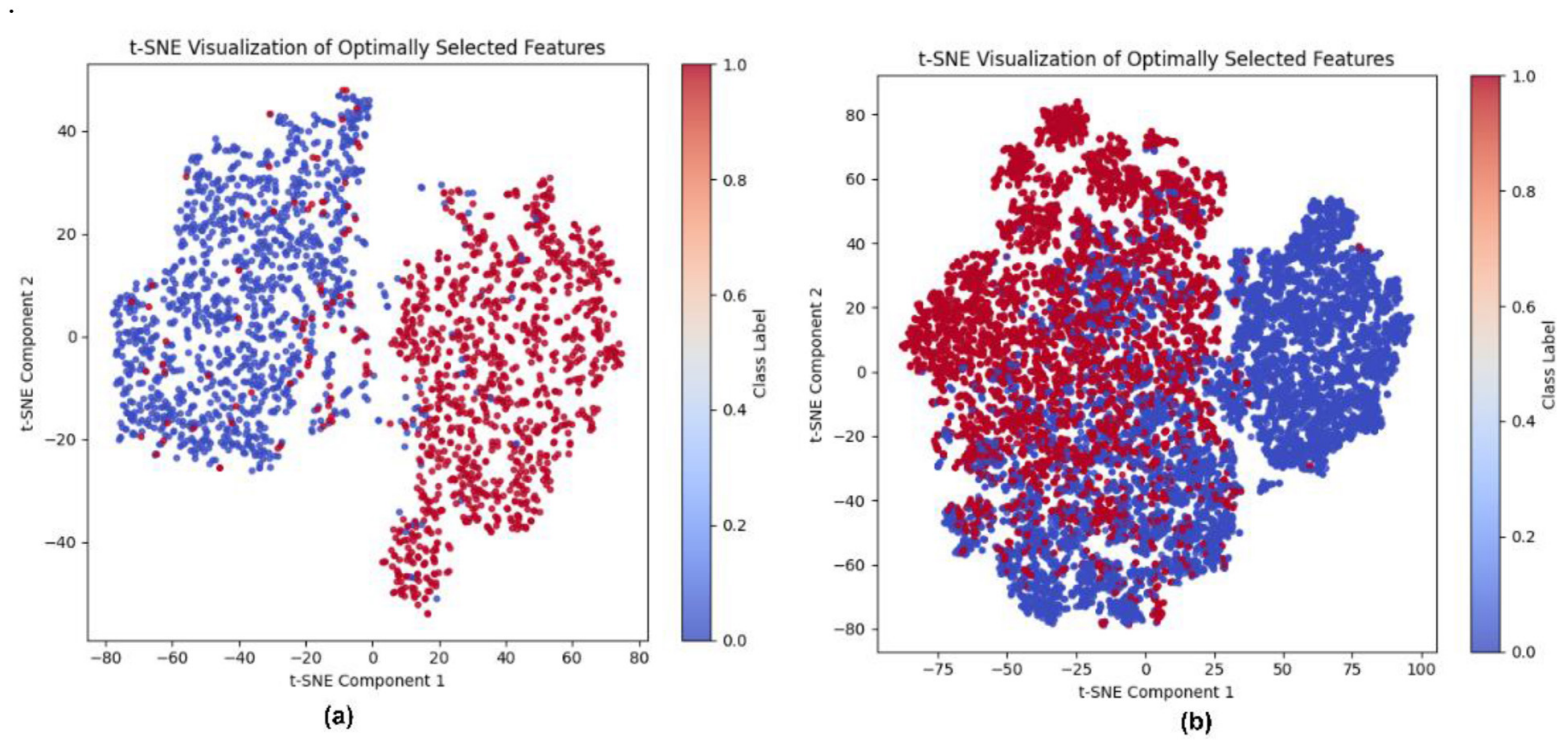
t-SNE visualization of optimal features for discriminating the class of both datasets: **(a)** PAD_UFES_20, **(b)** HAM10000.

t-SNE visualization projects high-dimensional characteristics into a two-dimensional space for qualitative examination. It illustrates the local connections among the samples by capturing and graphically representing the local structures. Each sample is shown as a point, with distinct color coding for individual labels. For skin cancer detection, there are two labels: benign and malignant, each represented by distinct colors. Figure 18 depicts the t-SNE for both datasets related to the binary classification problem, featuring selected attributes via the OFS approach.

The t-SNE visualization of PAD_UFES_20 clearly shows a separation of features with little overlap, indicating that the chosen features can discriminate among the class labels. In HAM10000, a greater degree of overlap is found compared to previous datasets, which may result from projecting high-dimensional characteristics onto a constrained two-dimensional representation; separation may be achievable through the hidden dimension.

### 4.8 Ablation study

An ablation study is performed to assess the impact of critical architectural components in the proposed framework. Table 7 compares performance metrics of various components of the architecture.

**Table 7:** Ablation study of OFS.

Methodology	PAD_UFES_20				HAM10000			
	Accuracy	Precision	Recall	F1-score	Accuracy	Precision	Recall	F1-score
Without OFS	70.65	71.06	70.65	70.28	84.43	84.09	84.43	84.25
Only with MI	87.17	87.85	87.17	87.17	90.97	90.45	90.97	90.45
Only with variance	78.26	78.75	78.26	78.26	84.03	84.35	84.03	84.18
Without redundancy	83.70	84.12	83.70	83.70	91.42	91.18	91.42	91.27
**Proposed (OFS)**	**93.26**	**93.38**	**93.26**	**93.25**	**95.66**	**95.83**	**95.66**	**95.72**

The bold values indicate higher performance of the proposed model compared to others.

### 4.9 Cross-dataset validations

Cross-dataset validation demonstrates the model's generalizability, robustness in real world scenario of the proposed model. The datasets used for experimentation PAD_UFES_20 and HAM10000 serves skin cancer images, but only four classes are in common. Two classes are common in benign type of lesions (AKIEC, NV) and two classes are common in malignant lesions (BCC, MEL). Two experiments are done: the first experiment is done by considering HAM10000 as train set and PAD_UFES_20 as test set and second experiment is done by considering PAD_UFES_20 as train set and HAM10000 as test set. Table 8 presents the experimental results of cross-dataset validations. There is a variation in the features selected by the SAMI method across two datasets. For cross-dataset validation experiments, the handcrafted features are selected based on the training set.

**Table 8:** Cross-dataset validations.

Dataset	Accuracy	Precision	Recall	F1-score	AUC
Train: PAD_UFES_20 Test: HAM10000	78.29	83.93	78.29	80.24	77.28
Train: HAM10000 Test: PAD_UFES_20	71.52	71.89	71.52	71.52	77.44

The bold values indicate higher performance of the proposed model compared to others.

### 4.10 Comparative study

**Figure 19:**
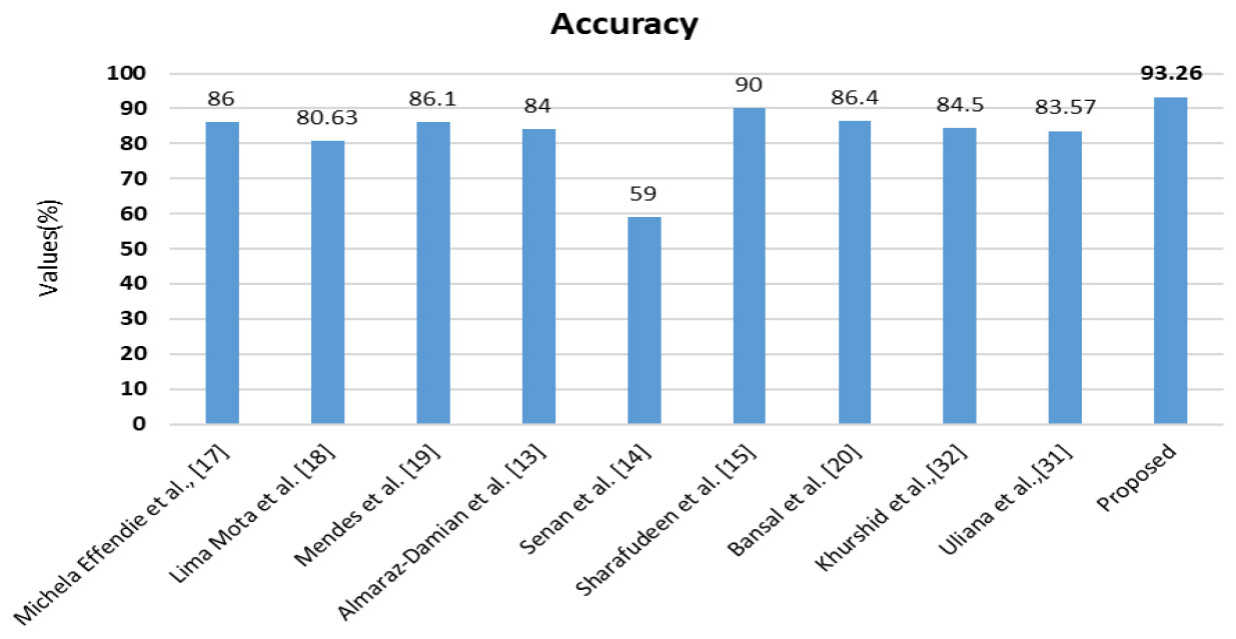
Comparative study of the performance of various previous works over the proposed model on PAD_UFES_20.

**Figure 20:**
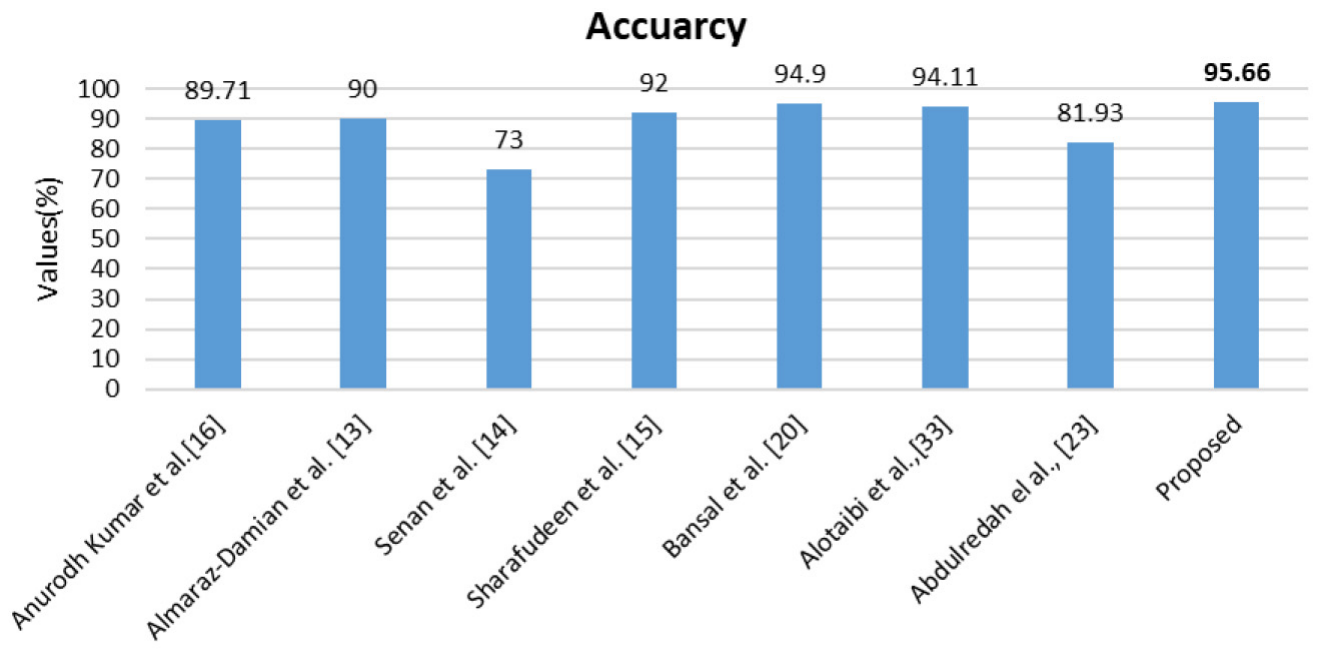
Comparative study of the performance of various previous works over the proposed model on HAM10000.

The comparison study demonstrates the superiority of the proposed approach, providing proof against other significant works. The extensive, densely packed performance metrics are employed for comparison analysis. This section of this study outlines two comparative methodologies for statistical measurements: one emphasizes comparison with state-of-the-art (SOTA) approaches, while the other contrasts them with prior publications. Tables 9, 10 illustrate the comparison between state-of-the-art machine learning models and statistical significance analysis with 95% CI. Figures 19, 20 illustrate the comparison between the proposed model and previous and recent studies, like (Uliana and Krohling, 2025; Khurshid et al., 2025; Alotaibi and AlSaeed, 2025). The results of the comparative study accurately elucidate the proposed model, which is appropriately calibrated against individual statistical metrics and existing literature.

**Table 9:** Comparative study of performance metrics of various ML models on PAD_UFES_20.

Methodology	Accuracy	Precision	Recall	F1-score	95% CI
SVM (linear)	79.35	79.63	79.35	79.21	75.41–82.80
SVM (quadratic)	81.30	81.50	81.30	81.21	77.49–84.60
SVM(RBF)	83.70	84.14	83.70	83.58	80.04–86.79
KNN	73.04	73.02	73.04	73.02	68.81–76.90
Random forest	77.39	77.61	77.39	77.25	73.35–80.98
**MLP(proposed)**	**93.26**	**93.38**	**93.26**	**93.25**	**90.59**–**95.21**

**Table 10:** Comparative study of performance metrics of various ML models on HAM10000.

Methodology	Accuracy	Precision	Recall	F1-score	95% CI
SVM (linear)	91.97	91.59	91.97	91.64	90.69–93.08
SVM (quadratic)	90.52	90.19	90.52	90.32	89.16–91.73
SVM (RBF)	91.12	90.76	91.12	90.88	89.79–92.29
KNN	84.88	85.09	84.88	84.98	83.25–86.38
Random forest	88.27	87.79	88.27	87.98	86.79–89.61
**MLP (proposed)**	**95.66**	**95.83**	**95.66**	**95.72**	**94.68–96.47**

### 4.11 Discussion and limitations

This study presents a technique for identifying the malignancy of skin cancer utilizing both handcrafted and non-handcrafted characteristics. The system enhanced detection accuracy by executing feature selection using two approaches, such as SAMI on handcrafted features and OFS on combined features.

This study presents an innovative way for extracting a certain handcrafted characteristic, the irregularity of the border of the skin lesion's form. This study also demonstrates that employing potential characteristics, such as Fitzpatrick skin type, enhanced the detection accuracy. An innovative approach by SAMI to identify prospective handmade components before their integration with non-handcrafted elements. This enhances the overall detection rate by eliminating superfluous features from a prospective collection of handcrafted features. The deep learning approach, which employs an innovative configuration of neural network layers, facilitates the acquisition of non-handcrafted features. At the end, OFS facilitates the aggregation of key statistical metrics and the refinement of hyperparameters for their weights to identify the top-k most informative detection features.

Detection performance can be further improved by enhancing the extraction process of handcrafted features. A few features are extracted from the binary mask of the skin lesion, such as asymmetry, border irregularity, and diameter. This highly depends on the accuracy of extracting the binary mask from the RGB images, especially when expert annotations are not available.

The performance of the proposed approach is evaluated by two datasets: HAM10000 is available with an expert-annotated binary mask, but PAD_UFES_20 doesn't have one. Binary masks for the unavailable dataset are produced using a conventional UNET model trained on images from the HAM10000 dataset, which then predicts binary masks for the PAD_UFES_20 dataset. The model attained a validation accuracy of 93.30%. Nevertheless, several photos of skin lesions with minor lesions are not recognized in the anticipated binary mask. An optimally segmented model may precisely identify the segmented masks of tiny and numerous lesions, thereby enhancing the feature capacity for malignancy detection.

## 5 Conclusion

The classification of skin lesions into benign and malignant categories is performed using both handcrafted and non-handcrafted features. Before feature extraction, all skin lesions undergo preprocessing to remove artifacts, including hair, thereby improving the quality of feature detection. A concurrent extraction of handcrafted features and non-handcrafted features is executed. The characteristics of the handcraft include six distinct features: asymmetry of lesion shape (A), irregularity of lesion border (B), variety of lesion colors (C), diameter of lesion shape (D), texture of lesion (T), and patient Fitzpatrick skin type (F). Asymmetry, border irregularity, and diameter characteristics are derived from the binary mask of the skin lesion, which delineates the form of the region of interest (ROI) of the lesion. The remaining non-handcrafted characteristics are derived from the DSRSENet model, which utilizes two parallel convolutional neural networks, referred to as shallow and deep networks, to extract features from skin lesion images.

A binary classification model is constructed to utilize both major handcrafted and non-handcrafted characteristics for optimal diagnosis of skin malignancy from skin lesion photos. The non-handcrafted features derived from deep neural network models exhibit reduced susceptibility to error and significantly enhance accurate categorization. Handcrafted features are more significant domain-wise; yet, they are more prone to errors owing to their extraction procedure, which relies on manual design and lacks dataset validation. Before transmitting handcrafted characteristics to the classification model alongside non-handcrafted features, a specialized feature selection method is employed to refine and retain just the most significant features that contribute substantially to categorization. SAMI is the feature selection approach employed for this assignment, utilizing mutual information to assess the importance of handcrafted features in relation to their contribution to classification and self-attention weights. Ultimately, features are picked based on both ranking and weighting criteria. When handcrafted and non-handcrafted features are concatenated for input to the classification model, a substantial duplication issue may arise, since both feature sets are derived from the same input images. To address this issue, an optimization technique is applied to the concatenated features, using a scoring fitness function and an accuracy objective function, while adjusting hyperparameters such as α, β, γ, and k to enhance model performance.

The assessment of the suggested models is conducted using the PAD_UFES_20 and HAM10000 datasets. Experimental results demonstrate that the proposed modifications in the extraction of border irregularity of skin lesions and the incorporation of Fitzpatrick skin type information in the detection of skin malignancy have resulted in an increase from 56.52% to 67.39% and from 83.92% to 84.27% for PAD_UFES_20 and HAM10000, respectively. The initial feature selection mechanism, SAMI, implemented on handcrafted features enhanced detection accuracy by pinpointing the most significant features contributing to detection, achieving 73.80% for PAD_UFES_20 and 89.42% for HAM10000. The DSRSENet model demonstrated effective performance in detection by extracting features, achieving a high accuracy of 79.35% for the PAD_UFES_20 dataset and 90.87% for the HAM10000 dataset. Finally, the application of OFS to a combination of selected handcrafted and non-handcrafted features resulted in an increased malignancy detection accuracy of 93.26% for PAD_UFES_20 and 95.66% for HAM10000.

### 5.1 Future plan

In the future, there is a desire to identify more relevant features for lesion malignancy detection and to extend the work to include multiclass classification in addition to utilizing these features for the prediction base.

## Data Availability

Data availability Two publicly available datasets are used for analysis the name and links as follows PAD_UFES_20 : https://data.mendeley.com/datasets/zr7vgbcyr2/1 HAM10000 : https://datasetninja.com/skin-cancer-ham10000.
